# The protective roles of integrin α4β7 and Amphiregulin-expressing innate lymphoid cells in lupus nephritis

**DOI:** 10.1038/s41423-024-01178-2

**Published:** 2024-05-28

**Authors:** Seungwon Ryu, Kyung Ah Kim, Jinwoo Kim, Dong Hun Lee, Yong-Soo Bae, Hajeong Lee, Byoung Choul Kim, Hye Young Kim

**Affiliations:** 1 Department of Microbiology, Gachon University College of Medicine, Incheon, 21999 South Korea; 2 Department of Nano-Bioengineering, Incheon National University, Incheon, 22012 South Korea; 3 Laboratory of Mucosal Immunology, Department of Biomedical Sciences, Seoul National University College of Medicine, Seoul, 03080 South Korea; 4 Department of Dermatology, Seoul National University College of Medicine, Seoul, 03080 South Korea; 5 Laboratory of Cutaneous Aging Research, Biomedical Research Institute, Seoul National University Hospital, Seoul, 03080 South Korea; 6 Institute of Human–Environment Interface Biology, Medical Research Center, Seoul National University, Seoul, 03080 South Korea; 7 Department of Biological Sciences, SRC Center for Immune Research on Non-lymphoid Organs, Sungkyunkwan University, Suwon, 16419 South Korea; 8 Department of Biological Sciences, Sungkyunkwan University, Suwon, 16419 South Korea; 9 Division of Nephrology, Department of Internal Medicine, Seoul National University Hospital and Seoul National University College of Medicine, Seoul, 03080 South Korea; 10 Institute of Allergy and Clinical Immunology, Seoul National University Medical Research Center, Seoul National University College of Medicine, Seoul, 03080 South Korea

**Keywords:** Innate lymphoid cells, Tissue residency, Adhesion molecules, Integrins, Kidney, Lupus nephritis, Amphiregulin, Innate lymphoid cells, Lupus nephritis, Chronic inflammation

## Abstract

Type 2 innate lymphoid cells (ILC2s) have emerged as key regulators of the immune response in renal inflammatory diseases such as lupus nephritis. However, the mechanisms underlying ILC2 adhesion and migration in the kidney remain poorly understood. Here, we revealed the critical role of integrin α4β7 in mediating renal ILC2 adhesion and function. We found that integrin α4β7 enables the retention of ILC2s in the kidney by binding to VCAM-1, E-cadherin, or fibronectin on structural cells. Moreover, integrin α4β7 knockdown reduced the production of the reparative cytokine amphiregulin (Areg) by ILC2s. In lupus nephritis, TLR7/9 signaling within the kidney microenvironment downregulates integrin α4β7 expression, leading to decreased Areg production and promoting the egress of ILC2s. Notably, IL-33 treatment upregulated integrin α4β7 and Areg expression in ILC2s, thereby enhancing survival and reducing inflammation in lupus nephritis. Together, these findings highlight the potential of targeting ILC2 adhesion as a therapeutic strategy for autoimmune kidney diseases.

## Introduction

Innate lymphoid cells (ILCs) serve as sentinels at the frontline of host defense and tissue homeostasis. Unlike adaptive immune cells such as T and B cells, ILCs do not express antigen-specific receptors; instead, they express constitutively expressed receptors that recognize cytokines and environmental signals [1], enabling them to respond quickly to changing microenvironments through cytokine production [2]. Based on their cytokine production and transcription factor expression, ILCs are categorized into three major groups, namely, ILC1s, ILC2s, and ILC3s. Each group has unique functions and specializes in different types of immune responses. Among ILCs, ILC2s are major producers of type 2 cytokines in nonlymphoid tissues [3, 4].

In the kidney, ILC2s play a crucial role in regulating inflammation and promoting tissue repair in various nephritis conditions, including renal ischemia‒reperfusion injury, adriamycin nephropathy, and lupus nephritis. These effects are essential for preventing renal damage. The activation and expansion of local ILC2s are initiated by cytokines such as IL-2, IL-25, and IL-33, which directly stimulate these cells [5]. Notably, the therapeutic potential of activated ILC2s is underscored by studies showing that the adoptive transfer of these cells can attenuate renal ischemia‒reperfusion injury in murine models [6–8]. These findings highlight the promising role of ILC2s in renal diseases. ILCs may also participate in immune-mediated renal injury in humans, since the number of peripheral blood ILCs in patients with lupus nephritis decreases as disease severity increases [9]. However, the precise roles of renal ILC2s in kidney homeostasis and disease development still require further elucidation.

Under homeostatic conditions, kidney ILC2s are located in both the cortex and medullary regions [10] and are often found in the adventitial cuffs around blood vessels but not inside vessels themselves [11, 12]. The mechanisms of the tissue retention and migration of kidney ILC2s under homeostatic or pathogenic conditions are not well understood. Most adult ILC2s originate from progenitor ILC2s (ILC2Ps) generated in the bone marrow and fetal liver and eventually settle in lymphoid and nonlymphoid organs. While settled ILC2s exhibit limited recirculation in the bloodstream and sustain their populations through self-renewal [13, 14], mature ILCs and ILCP-like cells in the blood can enter organs, and organ-resident ILCs can recirculate between organs *via* the bloodstream [15]. These migratory events are driven by various stimuli, including cytokines, chemokines, and pathogen-derived molecules. For example, the migration of ILC2Ps to the intestine is facilitated by the expression of the chemokine receptor CCR9 and integrin α4β7 [16], while helminth infection-induced migration of intestine-resident ILC2s to the lungs involves IL-25 stimulation and the expression of sphingosine 1-phosphate (S1P) receptors [17]. Similarly, the migration of bone marrow-derived ILC2s to the lung upon exposure to *Alternaria alternata* relies on the cell-surface integrin β1 [18]. Thus, the molecular mechanisms governing ILC migration may differ depending on the tissue of origin, the target tissue, and the disease context.

Lupus nephritis is a type of kidney inflammation commonly observed in systemic lupus erythematosus (SLE), an autoimmune disease in which antibodies target self-antigens such as double-stranded DNA (dsDNA) and RNA [19]. Here, we investigated the effect of adhesion molecules on renal ILC2s in two mouse models of lupus nephritis: the MRL-*lpr* model and the imiquimod (IMQ)-induced model. In healthy kidneys, renal ILC2s express high levels of integrin α4β7. However, during autoimmune kidney inflammation, Toll-like receptor 7/9 (TLR7/9) signaling in ILC2s reduces the expression of integrin α4β7, causing ILC2s to migrate out of the kidney. Our studies suggested that kidney ILC2s are retained in the kidney by adhering to VCAM-1, E-cadherin, and fibronectin on structural kidney cells. Decreased integrin α4β7 expression in ILC2s downregulates the production of cytokines, particularly Amphiregulin (Areg), which weakens the suppressive effect of ILC2s on local inflammatory T cells and promotes kidney inflammation. Conversely, IL-33 treatment upregulates integrin α4β7 and Areg expression in kidney ILC2s, leading to improved survival of lupus nephritis. Overall, targeting the adhesion molecules expressed by ILCs holds promise as an immunomodulatory approach for inflammatory diseases.

## Results

### The spontaneous development of lupus in MRL-*lpr* mice is associated with reduced kidney ILC2 numbers

**Fig. 1:**
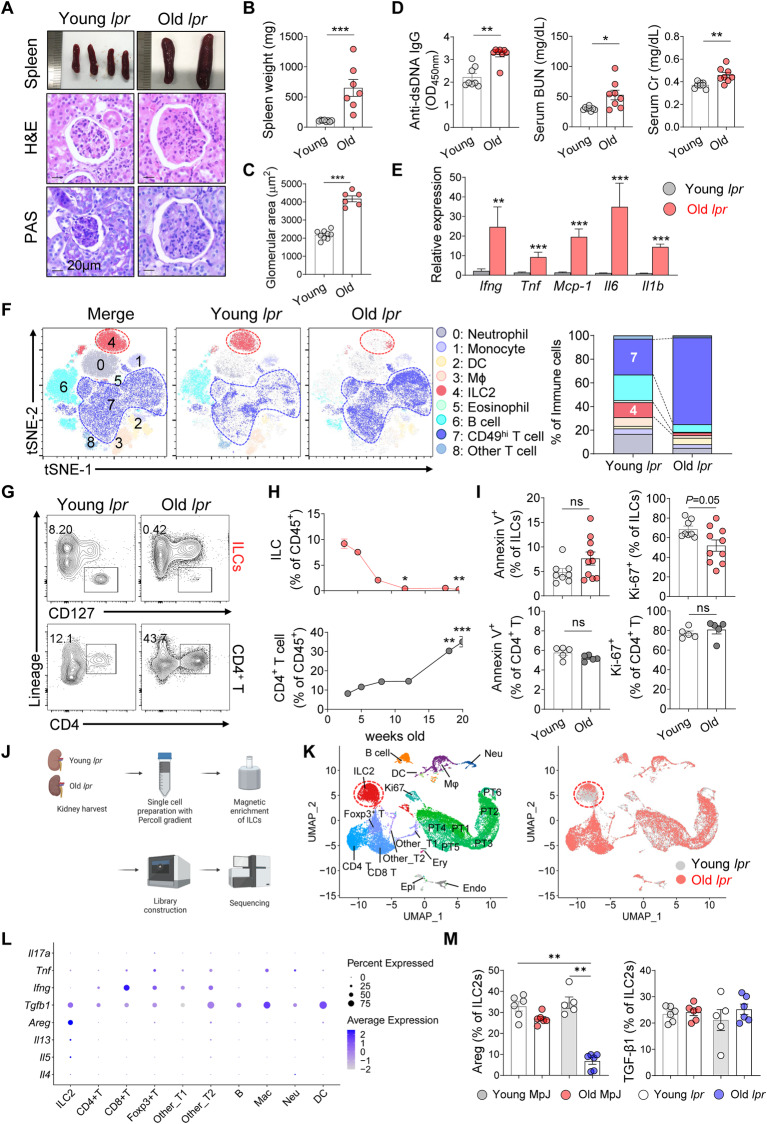
Reduced kidney ILC2 numbers linked to spontaneous lupus development in MRL-lpr mice. **A**–**C** Spleen morphology (**A**) and weight (**B**). Representative images of H&E and PAS staining showing the increased glomerular area in old MRL-*lpr* mice (**A**, **C**). Scale bars, 20 μm. **D** Serum anti-dsDNA IgG, BUN and creatinine (Cr) levels (*n* = 7–9). **E** Gene expression levels of *Ifng*, *Tnf*, *Mcp-1*, *Il6*, and *Il1b* in renal tissue were quantified using RT‒qPCR (*n* = 7). **F** Unbiased immunophenotyping of high-parameter flow cytometry data for CD45^+^ kidney immune cells. **G**–**H** Kidney ILCs (lineage^−^CD127^+^) and CD4^+^ T cells (lineage^+^ CD4^+^) (**G**) and their percentages among CD45^+^ immune cells (**H**) in 3- to 20-week-old MRL-*lpr* mice. (*n* = 4-5) (**I**) Flow cytometry analysis of Annexin V^+^ apoptotic cells and Ki-67^+^ proliferating cells among ILCs and CD4^+^ T cells (*n* = 8–10). **J** Experimental design for the scRNA-seq analysis of ILC-enriched renal cells from pooled samples from young and old MRL-*lpr* mice. **K** UMAP plot showing 20 distinct kidney cell types identified by unsupervised clustering (left panel) and their comparison across glomerulonephritis development in MRL-*lpr* mice (right panel). **L** Dot plot showing the expression of various inflammatory and regulatory cytokines in immune cell clusters. **M** Comparison of Areg and TGF-β1 expression assessed using flow cytometry (*n* = 5–6). All results are shown as the means ± SEMs, and the statistical analysis was performed using the Mann‒Whitney *U* test or Kruskal‒Wallis test. ns, not significant; *P < 0.05; **P < 0.01; and ***P < 0.001. DC dendritic cell, Mac or Mφ macrophage, Neu neutrophil, B B cell, T T cell, Ery erythrocyte, Epi epithelial cell, Endo endothelial cell, PT proximal tubule cell

We examined MRL-*lpr* mice, which spontaneously develop lupus-like disease, including glomerulonephritis, as they age [20] to study the behavior of ILC2s in renal inflammation. Although young (5–8-week-old) mice were not affected, old (>16-week-old) mice exhibited spleen enlargement; glomerular injury; elevated serum levels of anti-dsDNA, creatinine (Cr), and blood urea nitrogen (BUN) (Fig. 1A–D); and increased kidney expression of the inflammatory cytokines *Ifng*, *Tnf*, *Mcp-1*, *Il6*, and *Il1b* (Fig. 1E).

Renal CD45^+^ leukocytes obtained from young and old MRL-*lpr* mice were subjected to multicolor flow cytometry with representative markers of key immune cells (CD3ε, CD19, CD49b, CD11b, CD11c, Ly-6G, Ly-6C, F4/80, Siglec-F, FcεRIα, CD127, ST2, CD25, and CD45), and unsupervised clustering was performed to gain insights into the changes in the renal immune cell landscape that occur in lupus nephritis (Fig. S1A, B). The healthy kidney tissues from young MRL-*lpr* mice contained a robust population of ILC2s (defined as CD127^+^ ST2^+^ CD25^+^ leukocytes; Cluster 4) (Fig. 1F and Fig. S1A, B). In contrast, this population was significantly reduced in the old mice, and the T-cell cluster (Cluster 7) expanded instead (Fig. 1F and Fig. S1B). Conventional flow cytometry analysis confirmed a gradual decrease in kidney ILC2 frequencies and an increase in CD4^+^ T-cell frequencies as the disease progressed (Fig. 1G, H). Kidney ILCs in old mice exhibited less proliferation than those in young mice, but apoptosis remained unaffected (Fig. 1I). The proliferation and apoptosis of CD4^+^ T cells in the kidneys of the old mice did not change (Fig. 1I), but increased production of inflammatory cytokines, including IFN-γ and IL-17A, was observed (Fig. S1C). We also confirmed a reduction in the number of ILC2s and an increase in the number of CD4^+^ T cells in aged MRL-*lpr* mice compared to their background-matched strain, MRL-MpJ (Fig. S1D–H).

The functional changes in kidney ILC2s were then assessed via single-cell RNA sequencing (scRNA-seq). Given the low frequency of renal ILC2s in old mice, the cells from both old and young mice were first enriched with a magnetic kit (Fig. 1J). Principal component analysis (PCA) and uniform manifold approximation and projection (UMAP) analysis revealed 20 clusters of immune cells and nonimmune cells, including proximal tubule (PT) cells (Fig. 1K and Fig. S2A). The clusters included ILC2s and CD4^+^ T cells from both control and inflamed kidneys (Fig. 1K, right panel). The scRNA-seq analysis also revealed that kidney ILC2s expressed type 2 cytokine genes (*Il4*, *Il5*, and *Il13*), as well as *Areg* and *Tgfb1*, which are both known to have tissue-protective and immunoregulatory functions [21–24]. Notably, only ILC2s exhibited upregulated Areg expression, while T cells and other cell types produced TGF-β (Fig. 1L). Additionally, T cells exhibited higher expression of the pathogenic cytokine genes *Ifng*, *Tnf*, and *Il17a* than any other cluster (Fig. 1L). The flow cytometry analysis confirmed that the major immune cell population involved in Areg production was ILC2s (Fig. 1M and Fig. S2B). These results suggest that ILC2s may regulate the CD4^+^ T-cell-induced kidney inflammation observed in MRL-*lpr* mice.

### The inflamed kidneys of mice with IMQ-induced lupus exhibited reduced ILC2 numbers

**Fig. 2:**
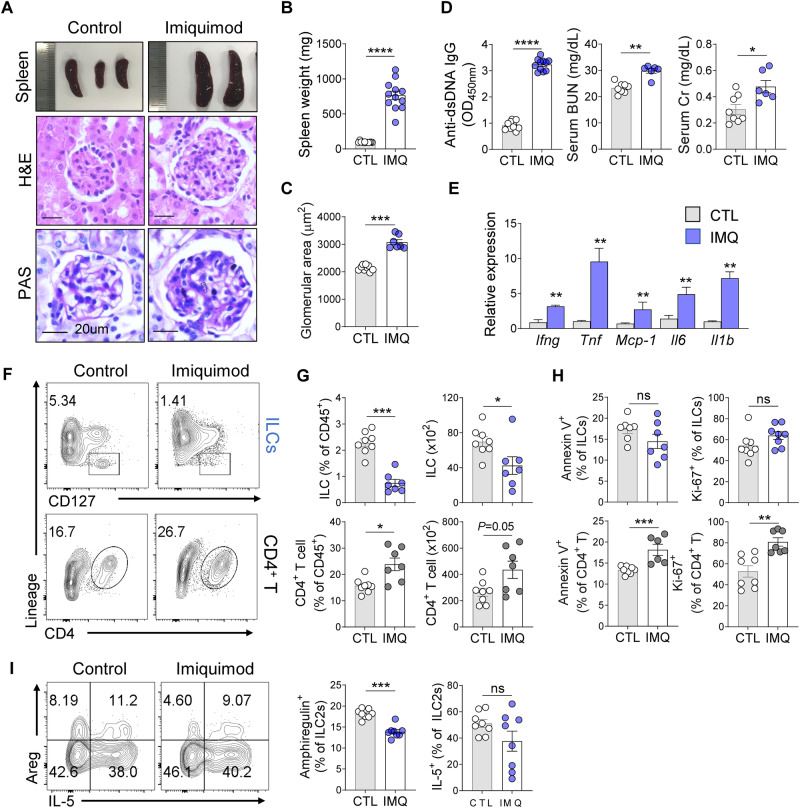
Decreased ILC2 frequencies in kidneys of mice with IMQ-induced lupus. **A**–**C** Spleen morphology (**A**) and weight (**B**) (*n* = 12). Representative images of H&E and PAS staining showing the increased glomerular area in the IMQ model (**A**, **C**; *n* = 7–9). Scale bars, 20 μm. **D** Serum anti-dsDNA IgG, BUN and creatinine (Cr) levels (*n* = 6–11). **E** Gene expression levels of *Ifng*, *Tnf*, *Mcp-1*, *Il6*, and *Il1b* in renal tissues were quantified using RT‒qPCR (*n* = 5). Kidney ILCs (lineage^−^CD127^+^) and CD4^+^ T cells (lineage^+^ CD4^+^) (**F**) and their percentages among CD45^+^ immune cells (**G**) in the IMQ model (*n* = 7–8). **H** Flow cytometry analysis of Annexin V^+^ apoptotic cells and Ki-67^+^ proliferating cells among ILCs and CD4^+^ T cells (*n* = 7). **I** Frequencies of AREG- and IL-5-expressing ILC2s in the IMQ model (*n* = 8). All results are shown as the means ± SEMs, and the statistical analysis was performed using the Mann‒Whitney *U* test. ns not significant, *P < 0.05; **P < 0.01; ***P < 0.001; and ****P < 0.0001

Repeated topical treatment of the inner ear skin of mice with the TLR7 agonist IMQ induces a lupus-like multiorgan autoimmune disease characterized by inflammation in the skin and kidneys [25–27]. We investigated renal pathology and immune cell populations in wild-type mice after 8 weeks of IMQ treatment to confirm our findings using MRL-*lpr* mice. The IMQ-treated mice also exhibited splenomegaly; glomerular injury; increased serum levels of anti-dsDNA, BUN, and Cr; and increased kidney expression of inflammatory cytokines (Fig. 2A–E). Moreover, the kidney ILC frequencies decreased significantly, while the kidney CD4^+^ T-cell frequencies increased (Fig. 2F, G). Importantly, the cell death and proliferation indices of renal ILCs did not differ between age-matched control and IMQ-treated mice, whereas CD4^+^ T cells exhibited significantly higher rates of apoptosis and proliferation (Fig. 2H). This result suggests that the loss of kidney ILC2s may be attributed to their migration out of the kidney. Notably, renal ILC2s from IMQ-treated mice exhibited reduced Areg expression, although no difference in IL-5 expression was detected (Fig. 2I).

### Kidney-resident ILC2s express high levels of integrin α4β7, and lupus nephritis is associated with the loss of ILC2s expressing this integrin

**Fig. 3:**
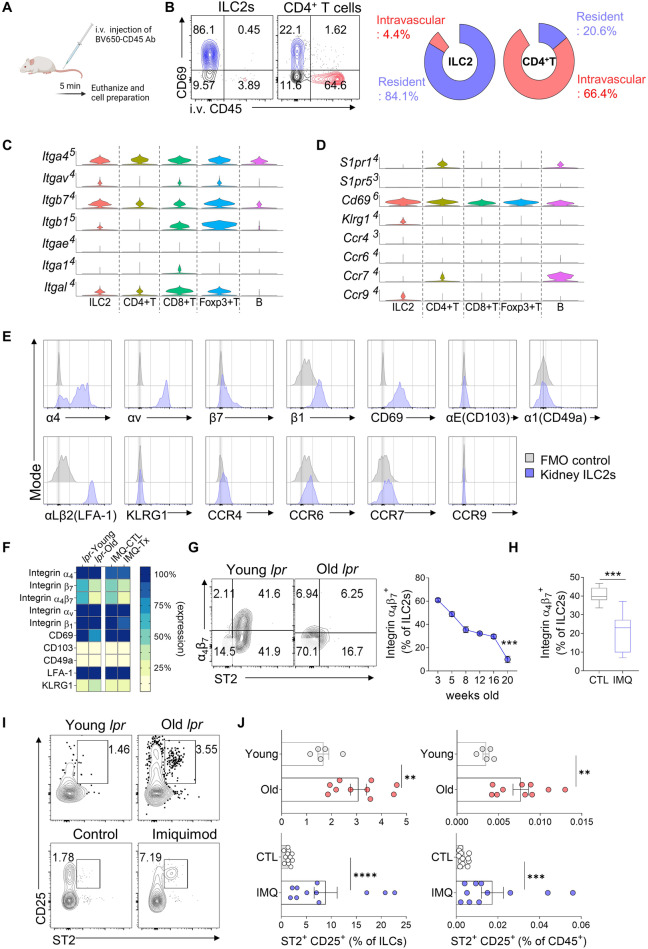
Loss of α4β7 expressing ILC2s in lupus nephritis. **A** Experimental design for intravascular immune cell staining following the tail vein injection of the BV650-CD45 monoclonal antibody. **B** Comparison of resident (CD69^+^ i.v.CD45^−^) and intravascular (CD69^−^ i.v.CD45^+^) cell compositions between ILC2s and CD4^+^ T cells in the naïve kidney (*n* = 4). Violin plot showing the expression of integrins (**C**) and other adhesion- and migration-related molecules (**D**) across lymphocyte clusters according to the scRNA-seq analysis. **E** Representative histogram of flow cytometry data for adhesion- and migration-related molecules evaluated in (**C**, **D**), excluding S1pr1 and S1pr5, overlaid with the FMO controls. **F** Heatmap showing the expression of adhesion molecules assessed using flow cytometry in the MRL-*lpr* and IMQ models, based on the frequency of expressing cells from Fig. 3**G**, **H** and Supplementary Fig. 3B, C. **G**, **H** Frequency of integrin α4β7-expressing kidney ILC2s in the MRL-*lpr* (**G**; *n* = 4–5) and IMQ lupus models (**H**; *n* = 8). **I**, **J** Representative flow cytometry plot (**I**) and frequency of splenic ST2^+^ CD25^+^ ILC2s in the MRL-*lpr* (*n* = 5–10) and IMQ lupus models (*n* = 11–12). All results are shown as the means ± SEMs, and the statistical analysis was performed using the Mann‒Whitney *U* test or Kruskal‒Wallis test. **P < 0.01, ***P < 0.001, and ****P < 0.0001

Naïve wild-type mice were injected with a BV650-labeled anti-CD45 antibody to investigate the tissue residency of ILC2s in healthy kidneys (Fig. 3A). Unlike CD4^+^ T cells, the majority of renal ILC2s did not exhibit BV650 fluorescence, indicating that they were tissue-resident cells. This finding was supported by the high expression of the tissue-resident marker CD69 [28] on ILC2s (Fig. 3B). The expression of genes encoding molecules involved in lymphocyte migration and adhesion [29–31] was assessed using our scRNA-seq data to gain a deeper understanding of the tissue residency of renal ILC2s (Fig. 3C, D). These cells expressed various integrins, such as *Itga4* (which encodes integrin α4), *Itgav* (integrin αv), *Itgb7* (integrin β7), *Itgb1* (integrin β1), and *Itgal* (integrin αL) (Fig. 3C). However, they did not express *Itgae* (integrin αE; also known as CD103) or *Itga1* (integrin α1; CD49a) (Fig. 3C). These cells also expressed *Cd69*, *Klrg1*, and *Ccr9* but lacked *Ccr4, Ccr6, or Ccr7* (Fig. 3D). The S1P receptors *S1pr1* and *S1pr5*, which have been implicated in ILC migration [17], were not expressed by kidney ILC2s (Fig. 3D). The flow cytometry analysis confirmed the expression of integrins α4, αv, β7, β1, and αLβ2, as well as CD69, by healthy kidney ILC2s (Fig. 3E). However, KLRG1, integrins αE and α1, CCR4, CCR6, CCR7, and CCR9 exhibited weak or no expression (Fig. 3E). Treatment with FTY720, an S1P receptor agonist, did not affect the frequency of kidney ILCs (Fig. S3A), supporting the absence of S1PR1 or S1PR5 expression (Fig. 3D).

In the lupus nephritis mouse models (MRL-*lpr* and IMQ models), a significant decrease in the expression of integrin α4β7 by kidney ILC2s was observed (Fig. 3F). This decrease was accompanied by a reduction in the frequency of ILC2s expressing integrin αv and integrin β1 in both models, but not in CD4^+^ T cells (Fig. S3B). Notably, the MRL-*lpr* mice showed a further decrease in integrin α4β7 expression as the disease progressed (Fig. 3G), and the IMQ-treated mice exhibited significantly lower integrin α4β7 expression at sacrifice (Fig. 3H). Additionally, kidney inflammation in MRL-*lpr* mice resulted in reduced expression of the tissue residency marker CD69 by kidney ILC2s (Fig. 3F and Fig. S3C). Thus, lupus nephritis is associated with the loss of integrin α4β7-expressing ILC2s in the kidney, possibly due to ILC2 migration from the inflamed kidney. This notion is supported by the fact that lupus nephritis increased the frequencies of ILC2s expressing CD25 and the IL-33 receptor ST2 in the spleen (Fig. 3I, J). Importantly, the α4β7-expressing kidney ILC2s were functional, as they did not express IL-18Rα, a marker of tissue ILCPs [32–34], but exhibited increased α4β7 expression upon IL-33 stimulation (Fig. S3D).

### Integrin α4β7 retrains kidney ILC2s through adhesion to VCAM-1, fibronectin, and E-cadherin

**Fig. 4:**
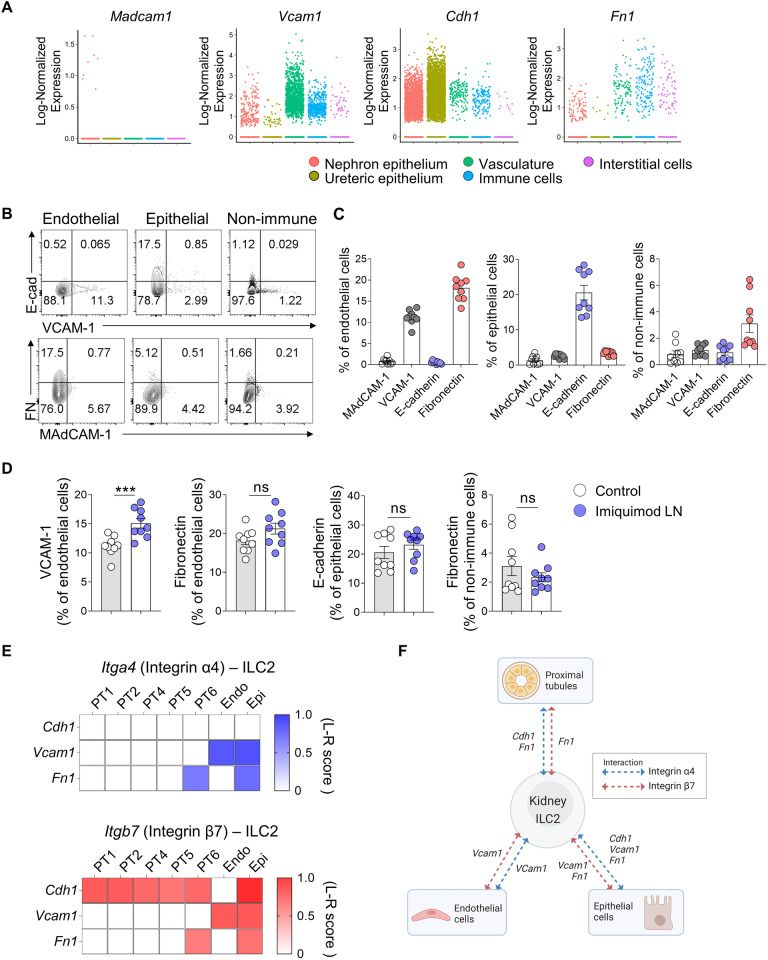
α4β7 integrin mediates kidney ILC2 retention. **A** Expression of *Madcam1*, *Vcam1*, *Cdh1*, and *Fn1* in various clusters of kidney cells from public mouse kidney scRNA-seq data assessed using KidneyCellExplorer (http://cello.shinyapps.io/kidneycellexplorer/). Representative flow cytometry plot (**B**) and frequencies (**C**) of MAdCAM-1, VCAM-1, E-cadherin, and fibronectin expression in endothelial (CD31^+^), epithelial (EpCAM^+^), and other nonimmune cells (CD45^−^ CD31^−^ EpCAM^−^) from the naïve mouse kidney (*n* = 9). **D** Expression of key adhesion molecules in the renal intrinsic cells from the IMQ model (*n* = 9). **E** Receptor‒ligand interactions between ILC2s and renal intrinsic cell clusters through *Itga4* (up) and *Itgb7* (down) expressed on ILC2s, as assessed by calculating the ligand‒receptor score (L-R score) from the analysis using the SingleCellSignalR algorithm. **F** Graphical summary showing the predicted interactions between ILC2s and renal intrinsic cells. All results are shown as the means ± SEMs, and the statistical analysis was performed using the Mann‒Whitney *U* test. ns not significant; ***P < 0.001. E-cad E-cadherin, FN fibronectin, Epi epithelial cells, Endo endothelial cells, PT proximal tubule cells

Publicly available scRNA-seq data from the KidneyCellExplore platform [35] were analyzed to identify the ligands in kidney tissue to which the integrins on kidney ILC2s adhere. *Vcam1, Cdh1* (which encodes E-cadherin), and *Fn1* (fibronectin) were found to be expressed by various kidney compartments, including the nephron epithelium, ureteral epithelium, blood vessels, and interstitial cells (Fig. 4A). However, *Madcam1* was expressed at very low levels in healthy renal tissue (Fig. 4A). Flow cytometry validation of mouse kidney tissues confirmed VCAM-1 and fibronectin expression on endothelial cells, E-cadherin expression on epithelial cells, and low levels of these ligands on other nonimmune cells, while MAdCAM-1 expression was absent (Fig. 4B, C).

We then assessed whether the expression of these ligands changed in the IMQ-induced lupus nephritis model. Endothelial cells were more likely to upregulate VCAM-1 in inflamed tissue, but the expression of fibronectin by endothelial cells and nonimmune cells and the expression of E-cadherin by epithelial cells were unchanged (Fig. 4D). This finding suggested that the migration of ILCs from the kidney was due to their downregulation of integrins rather than the downregulation of ligands by the kidney tissue. Considering the involvement of integrin α4β7 in ILC2 migration from the kidney (Fig. 3F–H), we utilized the SingleCellSignalR algorithm [36] to identify potential kidney ligands of *Itga4* (integrin α4) and *Itgb7* (integrin β7) and the corresponding ligand-expressing cells. The analysis suggested that epithelial cells expressed *Vcam1*, *Fn1*, and *Cdh1*; endothelial cells expressed *Vcam1*; and proximal tubule cells expressed *Fn1* and *Cdh1* (Fig. 4E). Thus, the maintenance of ILC2 residency in healthy kidneys is estimated to be largely mediated by integrin α4β7 binding to VCAM-1 on endothelial cells and epithelial cells and to fibronectin on epithelial cells and proximal tubule cells (Fig. 4F).

### Integrin α4β7-mediated adhesion and migration of renal ILC2s are mediated by their corresponding ligands

**Fig. 5:**
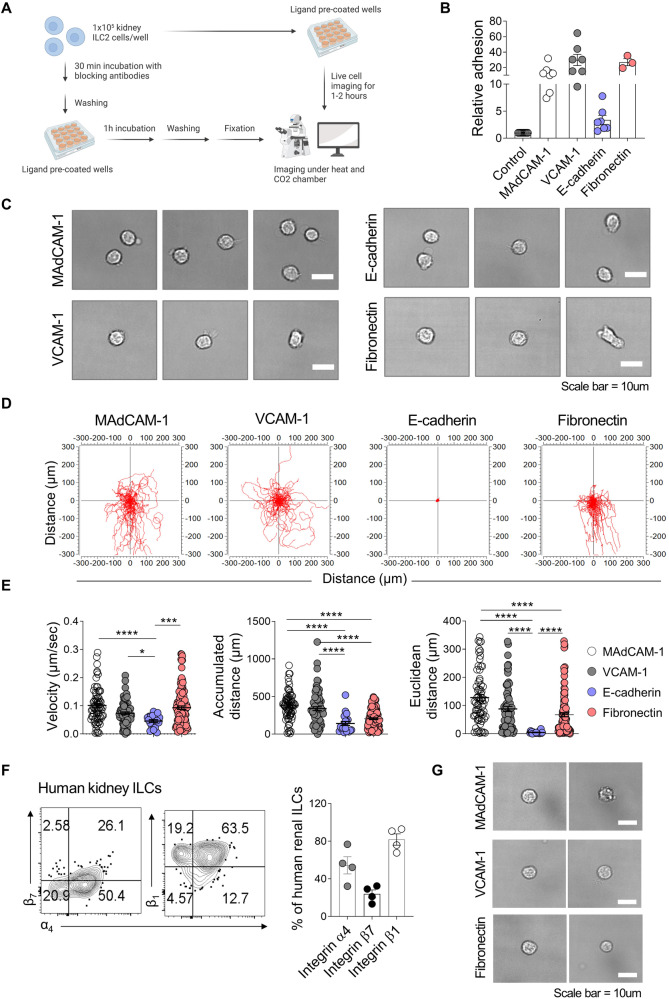
Integrin α4β7 guides renal ILC2 adhesion and migration. **A** Experimental design for evaluating the adhesion of kidney ILC2s to prospective ligands expressed on renal tissues using live cell imaging. **B** Relative adhesion of ILC2s to the ligands compared to the control group (control, *n* = 10; MAdCAM-1, VCAM-1, and E-cadherin, *n* = 7; fibronectin, *n* = 3). **C** Images of ILC2s adhered to ligand-coated plates. Scale bars, 10 μm. **D** Trajectories of the ILC2s tracked over time on ligand-coated plates. **E** Velocity, accumulated distance, and Euclidian distance of the ILC2 trajectory on ligand-coated plates. **F** Expression of integrin α4β7 and β1 in human kidney ILCs (*n* = 4). **G** Images of human blood ILCs adhered to ligand-coated wells. Scale bars, 10 μm. All results are shown as the means ± SEMs, and the statistical analysis was performed using the Kruskal‒Wallis test. ns not significant, *P < 0.05; ***P < 0.001; and ****P < 0.0001

The α4β7-mediated adhesion of healthy renal ILC2s to various ligands was then assessed *via* adhesion assays. Thus, the sorted renal ILC2s were incubated on ligand-coated plates and imaged under physiological conditions (Fig. 5A). The cells adhered most strongly to VCAM-1, followed by fibronectin and MAdCAM-1, while adhesion to E-cadherin was relatively weak (Fig. 5B). Live-cell imaging revealed that renal ILC2s displayed static adhesion to E-cadherin but exhibited vigorous adhesion to VCAM-1, fibronectin, and MAdCAM-1 (Fig. 5C, D, and Movies S1–4). The movement was fastest, and the cells ranged most widely from their starting point on MAdCAM-1 (Fig. 5E). These data, together with those in Fig. 4, suggest that integrin α4β7^+^ ILC2s can attach to renal endothelial cells *via* VCAM-1, to epithelial cells *via* VCAM-1 and fibronectin, and/or to proximal tubule cells *via* fibronectin. However, the ILC2s in the kidney are nonetheless able to bind to and migrate quickly on MAdCAM-1-expressing cells, which are absent in normal and lupus kidneys (Fig. 4B–D). While cell adhesion and movement may not always correspond to the same ligands, we hypothesized that cells adhering to any ligand contribute to the dynamic patrolling of ILC2s, thereby contributing to tissue immune homeostasis.

ILCs were sorted from normal kidney tissue obtained from patients who underwent resection for renal tumors and subjected to flow cytometry to test whether the same adherence mechanisms occur in normal human kidneys. Indeed, human kidney ILCs also expressed high levels of integrin α4, β1, and β7 (Fig. 5F), and human blood ILCs adhered to MAdCAM-1, VCAM-1, and fibronectin (Fig. 5G).

### TLR7 and TLR9 signaling reduces integrin expression in kidney ILC2s, whereas IL-33 stimulation increases integrin expression

We investigated the impact of TLR7 and TLR9 signaling on the expression of integrins in kidney ILC2s during inflammation, which is associated with the pathogenesis of autoimmune kidney diseases driven by autoantibodies [37, 38]. Initially, we expanded normal kidney ILC2s, and subsequently these cells were exposed to TLR agonists. While integrin α4 expression remained unchanged (Fig. S4A), the TLR7 agonist (ssRNA40) and the TLR9 agonist (ODN1826) reduced the expression of both integrin β7 and β1 (Fig. S4B, C). Thus, TLR7 and TLR9 signaling reduced ILC2 expression of integrins that may be involved in the retention of these cells in the kidney.

**Fig. 6:**
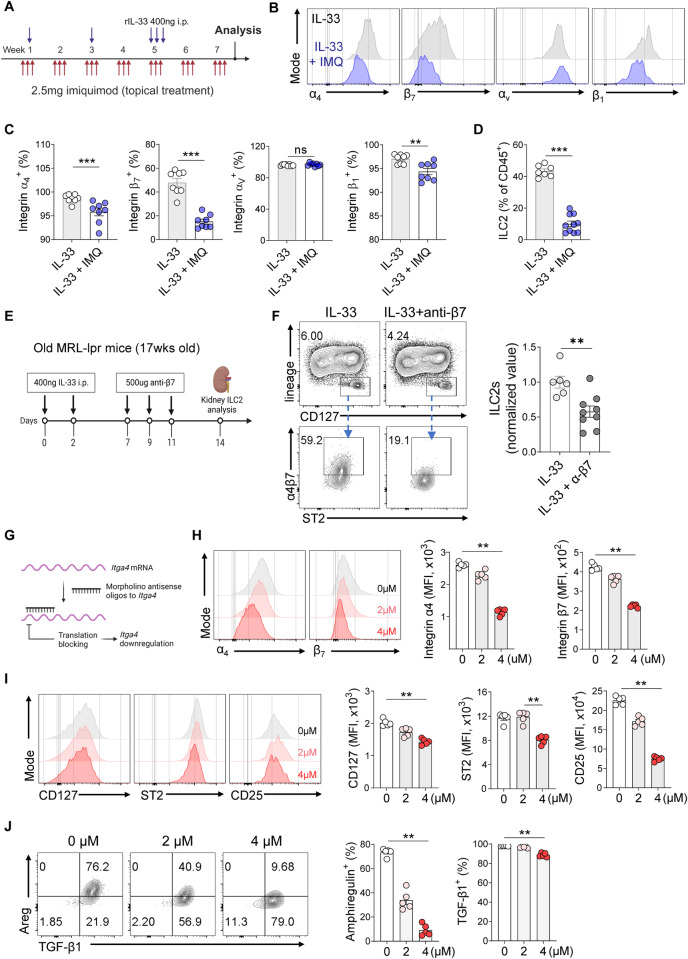
Reduced integrin expression in kidney ILC2s *via* TLR7/9. **A** Experimental protocol for in vivo TLR7 agonist treatment of IL-33-treated mice. Representative histograms (**B**) and frequencies (**C**) of integrin α4-, β7-, αv-, and β1-expressing ILC2s in IL-33-treated mice exposed to IMQ (*n* = 8). **D** Frequency of ILC2s in recombinant IL-33-treated mice exposed to IMQ (*n* = 8). **E** Experimental design for blocking integrin β7 in recombinant IL-33-treated old MRL-*lpr* (17 weeks old) mice. **F** Representative flow cytometry plot of ILCs and their frequencies after blocking integrin β7 in recombinant IL-33-treated old lpr mice (*n* = 6–9). **G** Graphical scheme of Itga4 MO-mediated downregulation of gene expression. **H** Expression (MFI) of integrin α4 and β7 after Itga4 MO transfection of kidney ILC2s (*n* = 5). **I** Expression (MFI) of CD127, ST2, and CD25 after Itga4 MO transfection of kidney ILC2s (*n* = 5). **J** Frequencies of AREG and TGF-β1 induced by Itga4 MO in kidney ILC2s (*n* = 5). All results are shown as the means ± SEMs, and the statistical analysis was performed using the Mann‒Whitney *U* test or Kruskal‒Wallis test. ns not significant; **P < 0.01; and ***P < 0.001

The administration of IL-33 to wild-type mice resulted in increased expression of integrins α4, β7, and β1 in renal ILC2s in vivo (Fig. S4D), suggesting a role for IL-33-ST2 in enhancing the expression of integrin α4β7 and opposing the effect of TLR7/9 signaling. We next asked whether TLR7 stimulation affected the upregulation of integrin expression by IL-33 (Fig. 6A). The flow cytometry analysis of kidney ILC2s revealed that IMQ treatment significantly reduced the IL-33-induced upregulation of integrin α4β7 expression in kidney ILC2s in vivo (Fig. 6B, C). Moreover, IMQ treatment significantly reduced the frequency of kidney ILC2s (Fig. 6D). Thus, IL-33 increased integrin expression on kidney ILC2s in vivo, while the TLR7 agonist IMQ reversed this effect and promoted the loss of ILC2s from the kidney.

We explored the role of the transcription factor LRF (encoded by *Zbtb7a*), considering potential parallels with T cells, where LRF has been associated with integrin α4β7 expression [39], to understand how TLR7 influences the downregulation of integrin α4β7 expression in ILC2s. Initially, we confirmed the expression of *Zbtb7a* in ILC2s using publicly available single-cell RNA sequencing data and noted that *Zbtb7a* was upregulated by IL-33, consistent with the expression patterns of *Itga4* and *Itgb7* (Fig. S4E). Notably, in the IMQ-induced model, we observed a significant decrease in LRF protein expression (Fig. S4F). Furthermore, upon treatment of lung ILC2s with ssRNA40, downregulation of *Itga4*, *Itgb7*, and *Areg* expression was observed concomitant with decreased *Zbtb7a* expression (Fig. S4G). Finally, we observed a positive correlation between the expression of LRF and integrin β7 in both control and IMQ-treated kidneys, providing further evidence for the proposed TLR7-LRF-integrin α4β7 axis in ILC2s during renal inflammation (Fig. S4H).

Old MRL-*lpr* mice were treated with IL-33 to expand the ILC2s, and an integrin β7-blocking antibody was administered to confirm that the disappearance of ILC2s from the kidney was driven by the loss of integrin α4β7 (Fig. 6E). The analysis of kidney ILC2s revealed that blockade of integrin β7 reduced the frequency of ILC2s in the kidney (Fig. 6F). Together, these findings provide further support for the notion that integrin α4β7 mediates the retention of ILC2s in the kidney and that TLR7/9 stimulation of ILC2s reduces the LRF-induced expression of integrin α4β7 in kidney ILC2s, consequently promoting ILC2 migration from the kidney.

### Integrin α4β7 knockdown in kidney ILC2s impairs cytokine and receptor expression

We used *Itga4*-targeting antisense morpholino oligonucleotides (MOs) [40] to specifically knock down integrin α4β7 in expanded ILC2s and investigate the role of integrin expression in kidney ILC2s in lupus nephritis (Fig. 6G). Intriguingly, as the MO concentration increased and integrin expression in the ILC2s decreased (Fig. 6H), the cells exhibited progressively lower levels of cytokine receptors that are crucial for ILC2 function, namely, CD127 (IL-7Rα), ST2 (IL-33R), and CD25 (IL-2Rα) [41–43] (Fig. 6I). This reduction in receptor expression correlated with decreased secretion of the conventional ILC2 cytokines IL-5 and IL-13 (Fig. S5A), as well as Areg and TGF-β1 (Fig. 6J). Notably, the impact on Areg secretion was particularly pronounced (Fig. 6J). We conducted similar experiments on CD4^+^ T cells from normal kidneys to assess the specificity of integrin α4β7 knockdown in ILC2s. Despite the reduction in integrin expression in CD4^+^ T cells, it did not affect the secretion of cytokines by these cells (Fig. S5B, C).

Notably, our scRNA-seq analysis revealed the expression of the tissue repair and regulatory cytokines Areg and TGF-β1 in renal ILC2s, with ILC2s serving as the primary cellular source of Aregs (Fig. 1L). While renal ILC2s are capable of alleviating inflammation through Areg secretion, persistent exposure to TLR stimulation in the context of autoimmune renal inflammation ultimately results in the downregulation of integrin α4β7 expression in ILC2s, leading to a decrease in Areg expression and the subsequent migration of ILC2s out of the kidney.

### IL-33-mediated expansion of kidney-resident ILC2s improves survival and renal function in models of lupus nephritis

**Fig. 7:**
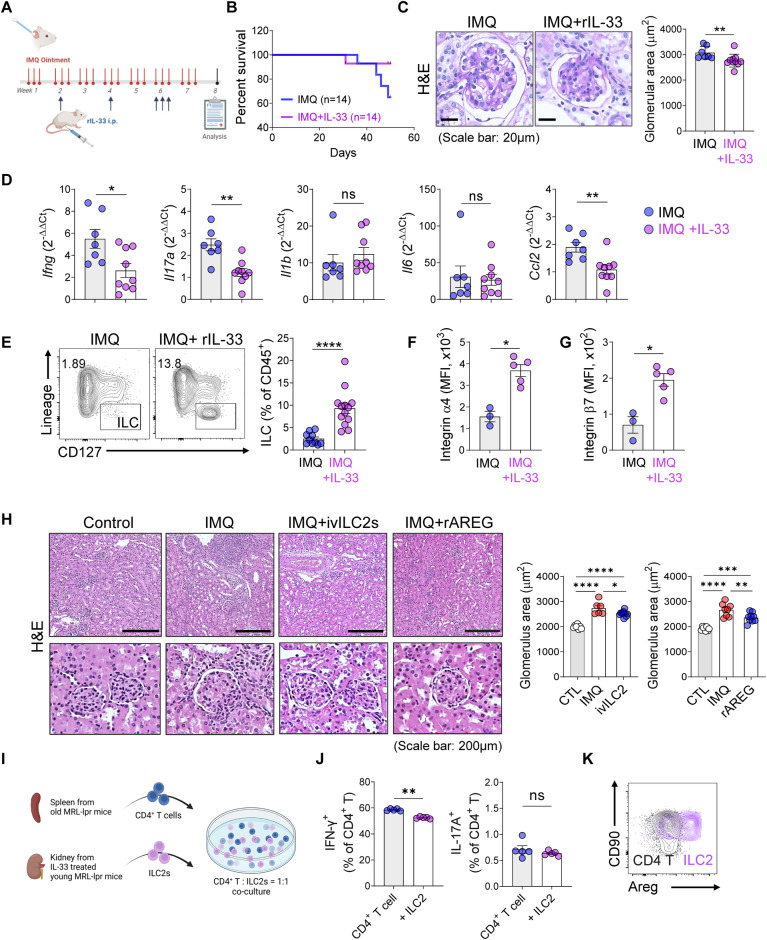
IL-33 enhances kidney ILC2s and renal function in lupus nephritis. **A** Experimental design of in vivo ILC2 expansion in the IMQ model. **B** Survival kinetics of the IL-33-treated IMQ model mice (*n* = 14). **C** Glomerular size in the IL-33-treated IMQ model mice was assessed using H&E staining (*n* = 7–10). **D** Gene expression levels of *Ifng, Il17a, Il1b, Il6*, and *Ccl2* in renal tissue were quantified using RT‒qPCR (*n* = 7‒9). **E** Frequencies of ILCs in the IMQ model mice after IL-33 treatment (*n* = 10–13). **F**, **G** Flow cytometry analysis of the expression of integrin α4 (**F**) and β7 (**G**) in kidney ILC2s (*n* = 3–5). **H** Glomerular size in the ILC2 adoptive transfer (ivILC2) or rAREG treated IMQ model mice was assessed using H&E staining (*n* = 6–11). **I** Experimental design of the in vitro assay in which splenic CD4^+^ T cells from old MRL-*lpr* mice and IL-33-treated kidney ILC2s from young MRL-*lpr* mice were cocultured. **J** Frequencies of IFN-γ and IL-17A in splenic CD4^+^ T cells from old MRL-*lpr* mice cocultured with IL-33-treated kidney ILC2s from young MRL-*lpr* mice. **K** Comparison of Areg expression between ILC2s and CD4^+^ T cells from cocultures (*n* = 5). All results are shown as the means ± SEMs, and the statistical analysis was performed using the Mann‒Whitney *U* test or Kruskal‒Wallis test. ns not significant; *P < 0.05; **P < 0.01; and ****P < 0.0001

IL-33 treatment induced the upregulation of integrin α4β7 expression in kidney ILC2s (Figs. S3D and S4D), suggesting a potential role for IL-33 in enhancing ILC2 retention in the kidney. Building on our previous findings implicating integrins in ILC2 renal retention and lupus nephritis pathogenesis, we aimed to investigate the potential of IL-33 treatment to improve the progression of lupus nephritis (Fig. 7A). The results showed that IL-33 treatment improved the survival of IMQ-treated mice (Fig. 7B). Although it did not significantly reduce spleen weight or anti-dsDNA levels (Fig. S6A, B), IL-33 treatment ameliorated kidney inflammation, as evidenced by decreased serum creatinine levels (Fig. S6C), a reduced glomerular size in the IL-33-treated IMQ group (Fig. 7C), and decreased tissue levels of proinflammatory cytokines (IFN-γ, IL-17A, and CCL2) (Fig. 7D). Furthermore, IL-33 treatment was associated with an increased frequency of ILCs, but not Tregs or macrophages, in the kidney (Fig. 7E and Fig. S6D, E), accompanied by elevated expression levels of the integrins α4 and β7 (Fig. 7F, G).

We transferred ILC2s and administered recombinant Areg (rAreg) to IMQ-treated mice to validate the direct role of ILC2s and their production of Areg. Due to the limited availability of kidney ILC2s, lung ILC2s were utilized following confirmation of their Areg expression. In the ILC2 transfer and recombinant Areg treatment experiments, the systemic parameters of autoimmune diseases remained unaffected by the treatments (Fig. S7A, C). However, subtle alterations were observed in kidney function markers, including serum BUN and Cr levels (Fig. S7B, D). Notably, tissue inflammation and the glomerular area were significantly reduced by both ILC2 transfer and rAreg treatment (Fig. 7H). These findings, in addition to those of IL-33 treatment experiments, provide direct evidence supporting the Areg-dependent regulation of kidney inflammation mediated by ILC2s.

Considering the tissue-regenerative and inflammation-suppressive properties of Areg, we aimed to investigate whether Areg secreted by ILC2s could regulate the activity of T cells involved in renal inflammation. We cocultured splenic CD4^+^ T cells isolated from old MRL-*lpr* mice, which exhibit elevated levels of inflammatory cytokines, with IL-33-treated renal ILC2s from young MRL-*lpr* mice to address this issue (Fig. 7I). This coculture resulted in a decrease in inflammatory cytokine production by CD4^+^ T cells (Fig. 7J). Notably, Areg production was predominantly attributed to ILC2s rather than to CD4^+^ T cells under these coculture conditions (Fig. 7K). This result further supports the notion that targeting the IL-33-ILC2 pathway and harnessing Areg production could have therapeutic potential for controlling renal inflammation.

## Discussion

In this study, we revealed the pivotal role of ILC2 adhesion in maintaining renal homeostasis. Healthy kidney ILC2s maintain their residency in the kidney by adhering to renal-intrinsic cells *via* integrin α4β7 binding to VCAM-1, fibronectin, and E-cadherin. However, in the context of lupus nephritis, the downregulation of integrin α4β7 on renal ILC2s, which is induced by TLR7/9 activation, disrupts their adhesion to the kidney and promotes their migration to the circulation. Notably, the loss of integrin α4β7 signaling in renal ILC2s also reduces the production of cytokines, particularly Areg. As lupus nephritis progresses, the decreased expression of integrin α4β7 on renal ILC2s leads to a decrease in Areg production, potentially impairing the ability of these cells to suppress the production of inflammatory cytokines by renal CD4^+^ T cells and contributing to renal inflammation. However, treatment with exogenous IL-33 restores integrin α4β7 expression in ILC2s, mitigating these effects. Our findings revealed a novel TLR7/9/IL-33-integrin α4β7 signaling mechanism that regulates the maintenance and activity of tissue-resident ILC2s in the kidney microenvironment.

The possibility that lupus nephritis is associated with the migration of ILC2s from the kidney is based on our in vivo model. We observed a significant decrease in kidney ILC2 numbers in mice with IMQ-induced lupus nephritis compared to those in age-matched control mice. However, the proliferation and apoptosis indices of kidney ILC2s in lupus nephritis mice were similar to those in the control group. In the MRL-*lpr* model, we also observed a decrease in kidney ILC2 numbers as the disease progressed compared to those in age- and background-matched MRL-MpJ mice.

The reduced expression of integrin α4β7 seemed to mediate the loss of kidney ILC2s in lupus nephritis in our study. Although adhesion molecules are well known for their involvement in lymphocyte attachment and cell-to-cell interactions in tissue homeostasis and inflammation [44, 45], little is currently known about their roles in ILCs. One study showed that integrin β2 expression mediates the trafficking of bone marrow-derived ILC2s to the lung and that this trafficking process is upregulated by allergen challenge [18]. However, the specific mechanisms involved are poorly understood, and even less is known about the role of adhesion molecules in ILC behavior in homeostatic or inflamed kidneys. Thus, our findings provide new insights for this field. Our algorithmic analysis of single-cell RNA sequencing data, flow cytometry data, and adhesion assays also suggested that the retention of ILC2s in healthy kidneys is mediated by their integrin α4β7-mediated attachment to VCAM-1 on endothelial cells, VCAM-1 and fibronectin on epithelial cells, and/or fibronectin on proximal tubule cells. Interestingly, live-cell imaging revealed that kidney ILC2s moved rapidly and over long distances when they were cultured on VCAM-1 or fibronectin. This dynamic ILC2 behavior has also been observed in vivo using ILC2s from lung and skin tissue and was reported to be due to their interactions with extracellular matrix components such as collagen and fibronectin [46, 47]. This dynamic behavior suggests that kidney ILC2s actively patrol local sites.

TLR signaling originating from the microenvironment of lupus nephritis may directly regulate the expression of integrin α4β7 in kidney ILC2s. Our study showed that TLR7/9 signaling, which is upregulated in autoimmune kidney inflammation, suppressed integrin α4β7 expression, while exogenous IL-33-mediated signaling increased the expression of this integrin. Notably, two previous studies have shown that *Salmonella* or viral infection downregulates integrin α4β7 expression in CD8^+^ T cells in the gut by inducing TLR7/9 signaling in these cells [48, 49]. This finding suggests potential conservation of the mechanisms regulating integrin α4β7 expression between ILC2s and CD8^+^ T cells. After considering this shared phenomenon, we hypothesized that LRF may contribute to integrin α4β7 downregulation in ILC2s. Nie et al. reported that the transcription factor LRF promotes the expression of integrin α4β7 in CD8αα^+^ intraepithelial lymphocyte precursors [39]. They elucidated that LRF binds to the *Itgb7* locus, thereby modulating its upregulation. We also found that LRF expression was downregulated by lupus conditions in vivo and the TLR7 agonist ssRNA40, suggesting shared mechanisms involving TLR7-LRF-integrin α4β7 signaling. However, different machinery may govern integrin α4β7 expression in CD4^+^ T cells, as our results showed its upregulation during lupus nephritis.

Notably, our study revealed a significant impact of integrin α4β7 expression on the function of kidney ILC2s. Specifically, we observed that downregulation of integrin α4β7 using *Itga4* MO led to a substantial reduction in the production of cytokines by ILC2s in vitro, particularly Areg. This finding highlights the crucial role of integrin α4β7 in modulating the functional capabilities of kidney ILC2s, which has not been reported previously. However, the precise mechanisms by which integrin α4β7 influences Areg expression and its associated signaling pathways remain elusive. Understanding these intricate mechanisms will provide valuable insights into the regulation of Areg production in kidney ILC2s and its potential impact on tissue repair and immune modulation.

Previous studies have shown that adoptive transfer or IL-33-mediated expansion of ILC2s can reduce inflammation in the kidney [6–8]. Our scRNA-seq analysis revealed that ILC2s are the primary immune cell type expressing *Areg* in renal tissue. Areg, a ligand for the epidermal growth factor receptor, is produced by various cell types, including epithelial, mesenchymal, and immune cells. Areg is renowned for its capacity to promote tissue repair during inflammation and to reduce inflammation [50]. Studies in the lung and intestine have highlighted the role of ILC2s in the secretion of Areg, which contributes to epithelial cell repair [21, 22]. Additionally, our coculture experiment indicated that IL-33-stimulated kidney ILC2s from young MRL-*lpr* mice produced significant amounts of Areg and directly reduced the production of inflammatory cytokines by T cells from old MRL-*lpr* mice. Crucially, treatment of IMQ-induced lupus mice with Areg resulted in a reduction in the glomerular area and inflammation. This finding is consistent with a recent study reporting severe pristane-induced lupus nephritis in *Areg* knockout mice [23]. Thus, a plausible hypothesis is that kidney ILC2s produce Areg during lupus nephritis to promote tissue repair and suppress local inflammation.

Finally, we showed that treatment with IL-33, which upregulates both integrin α4β7 and Areg expression in kidney ILC2s, led to reduced mortality in the IMQ-induced lupus nephritis model. This protective effect correlates with diminished renal inflammation and an increased presence of renal ILC2s. Furthermore, IL-33 treatment of IMQ-treated mice decreased the tissue levels of inflammatory cytokines, which was primarily attributed to T cells, according to our scRNA-seq data. Although IL-33 treatment induces the expansion of other IL-33 receptor-expressing immune cells, such as Tregs and alternatively activated macrophages, our findings emphasize the dominant expansion of ILC2s by IL-33 and indicate that ILC2s reduce renal inflammation. These findings align with previous studies highlighting the protective effects of IL-33 treatment or adoptive transfer of ILC2s on glomerulonephritis [6–8]. Hence, integrin α4β7, particularly in conjunction with its downstream product Areg, plays a pivotal role in the renal protection conferred by kidney ILC2s.

In summary, our study highlights the crucial involvement of integrin α4β7 in mediating the tissue retention and functional capacity of ILC2s, including their ability to produce Areg. The regulation of integrin α4β7 expression by TLR signaling within the kidney represents a promising avenue for developing novel therapeutic approaches targeting lupus nephritis and potentially other autoimmune kidney diseases. Strategies aimed at transferring appropriately adherent ILC2s or increasing the number of kidney-resident ILC2s also hold potential as cell-based therapies. Further research in this area will be instrumental in advancing our understanding and developing effective interventions.

## Materials and methods

### Mouse strains

MRL-MpJ and MRL-*lpr* (MRL/MpJ-*Fas*^*lpr*^/J) mice were obtained from The Jackson Laboratory (CA, USA) and Japan SLC (Shizuoka, Japan). BALB/c mice were acquired from Koatech (Gyeonggi-do, Korea). Female mice were used for all experiments, unless specified otherwise. All mice were bred and maintained under specific pathogen-free conditions at the Seoul National University Hospital Biomedical Research Institute and Gachon University Lee Gil Ya Cancer and Diabetes Institute Mouse Facility.

### Human participants

Human kidney tissues were obtained from patients with renal cell carcinoma (*n* = 3) and urothelial carcinoma (*n* = 1) who underwent nephrectomy. Only tissues without pathological changes were included in the analysis. Human blood samples were collected from healthy volunteers.

### Murine models of lupus nephritis

In the MRL-*lpr* spontaneous lupus mouse model, 5–8-week-old MRL-*lpr* female mice (Young *lpr*) were used as a control or mildly diseased group, while >16-week-old female MRL-*lpr* mice (Old *lpr*) served as the severely diseased group. MRL-MpJ mice of corresponding ages were used as background control mice for MRL-*lpr* mice. In the IMQ-induced model, lupus nephritis was generated by IMQ treatment according to previously described methods [25–27]. Briefly, the inner ears of 6–8-week-old female BALB/c mice were epicutaneously treated with 2.5 mg of IMQ cream (Aldara^TM^ cream, Donga ST) three times a week for 7 weeks. Age-matched untreated mice served as a control group. The mice were euthanized at week 8, unless specified otherwise. Systemic autoimmunity and renal involvement in the model mice were monitored by collecting blood, spleen and kidney samples before and after euthanasia.

### Tissue preparation and cell isolation

Mice were euthanized under isoflurane anesthesia. Whole kidneys were harvested and stored in cold PBS for less than 30 min until further processing. The tissues were then dissociated into small pieces using razor blades and incubated with digestion solution (collagenase IV (1 mg/ml; Worthington, LS004189) and DNase I (50 µg/ml; Sigma, DN25)) in RPMI 1640 media (Welgene, L0948-500) supplemented with 10% FBS (Biowest, S1480) for 30 min at 37 °C. Single cells were obtained by filtering the solution through 40 μm strainers. Renal leukocytes were further enriched by density-gradient separation. Total kidney cells were resuspended in a 40% Percoll solution (GE Healthcare, 17-0891-01) and layered on top of an 80% Percoll solution. After centrifugation at 2000 rpm for 30 min, cells were collected from the layer between the 40 and 80% Percoll solutions. Red blood cells were removed using RBC lysis buffer (Biolegend, 420302), and the cells were washed with FACS buffer (2% FBS in PBS) for use in further experiments.

### In vivo TLR7 agonist treatment in an IL-33-treated model

Recombinant IL-33 (400 ng; Biolegend, 580508) was administered intraperitoneally at weeks 1, 3 and 5. For the IMQ and IL-33 cotreatment group, 2.5 mg of IMQ was administered three times a week for 8 weeks.

### Integrin β7-blocking antibody treatment of MRL-*lpr* mice

Seventeen-week-old MRL-*lpr* mice received a treatment regimen consisting of 400 ng of recombinant IL-33 on Days 2 and 4, followed by the administration of 500 µg of an integrin β7-blocking antibody (BioXCell, BE0062) on Days 7, 9, and 11. Kidney tissues were harvested on Day 14 for the evaluation of tissue-resident ILC2 numbers.

### Recombinant IL-33 treatment of the IMQ-induced model

IMQ-induced model mice were treated with recombinant IL-33 (400 ng) in Weeks 2 and 4, and three times in Week 6. The mice were euthanized at Week 8 for further analysis.

### Adoptive transfer of ILC2s in the IMQ-induced model

ILC2s isolated from lung tissues were cultured and expanded in complete RPMI 1640 medium supplemented with 10 ng/ml IL-2 (BioLegend, 575404), 10 ng/ml IL-7 (BioLegend, 577804), and 30 ng/ml IL-33 (BioLegend, 580508). Subsequently, 1 × 10^6^ ILC2s were transferred on the first day of Weeks 2, 4, and 6. The mice were euthanized at the end of Week 6 for further analysis.

### Recombinant amphiregulin treatment of the IMQ-induced model

Recombinant amphiregulin (10 μg; R&D Systems, 989-AR-100/CF) was administered twice weekly for 5 weeks in the IMQ-induced model. The mice were euthanized at the end of Week 6 for further analysis.

### Flow cytometry analysis

Single cells were initially stained with Zombi-Aqua (BioLegend, 423102) to exclude dead cells from the analysis. Subsequently, the cells were incubated with Fc receptor blocking reagent (anti-mouse antibody from Biolegend (101320) or anti-human antibody from BD Biosciences (564219)) for 10 min on ice. The cells were further stained for 30 min with combinations of fluorochrome- or biotin-conjugated monoclonal antibodies (all from Biolegend, unless indicated otherwise) against mCD45 (clone: 30-F11), mCD11b (M1/70, BD Biosciences), mCD11c (HL3, BD Biosciences), mCD19 (1D3/CD19), mCD3ε (145-2C11), mCD49b (DX5), mF4/80 (BM8), mCD127 (A7R34), mCD90.2 (30-H12), mIL-33R (RMST2-33, Invitrogen), mCD25 (PC61), mCD49d (9C10), mCD51 (RMV-7), h/m integrin β7 (FIB504), mCD29 (HMβ1-1), mLPAM-1 (DATK32), mCD69 (H1.2F3), mCD103 (2E7), r/m CD49a (Ha31/8, BD Biosciences), mCD11a/CD18 (H155-78), h/m KLRG1 (2F1/KLRG1), mCD194 (2G12), mCD196 (29-2L17), mCD197 (4B12), mCD199 (9B1), mCD218a (A17071D), mCD4 (GK1.5), mCD31 (390), CD326 (G8.8), CD106 (429 (MVCAM.A)), h/m CD324 (DECMA-1), mMAdCAM-1 (MECA-367), fibronectin (F14, abcam), hCD45 (HI30), hCD11b (ICRF44), hCD11c (3.9), hCD14 (HCD14), hCD19 (HIB19), hCD3 (UCHT1), hCD49b (P1E6-C5), hFcεRIα (AER-37 (CRA-1)), hCD127 (A019D5), hCD49d (9F10), and hCD29 (HMbeta1-1). For biotin-conjugated antibodies, the cells were also stained with streptavidin PerCP-Cy5.5 (BioLegend, 405214) for 30 min on ice and washed with FACS buffer. For Annexin V staining, the cells were resuspended and incubated with Annexin V APC (BioLegend, 640920) in Annexin V binding buffer for 15 min on ice and analyzed within 30 min without a washing step. The cells were restimulated with 100 ng/ml PMA (Sigma), 1 µg/ml ionomycin (Sigma), and 1 µl/ml protein transport inhibitor (Golgi Plug, BD Biosciences) in RPMI 1640 medium supplemented with 10% FBS for 4 h to evaluate cytokine production. Intracellular staining was conducted by fixing and permeabilizing cells using either a Foxp3/Transcription Factor Staining Kit (Invitrogen, 00-5523-00) or a Cytofix/Cytoperm Kit with Golgi Stop (BD Biosciences, 554715) according to the manufacturer’s instructions. The fixed and permeabilized cells were then incubated for an hour with fluorochrome-conjugated monoclonal antibodies (all from Biolegend, unless indicated otherwise) against h/m T-bet (4B10), mGATA-3 (L50-823, BD Biosciences), mRORγt (Q31-378, BD Biosciences), mKi-67 (16A8), h/m LRF (13E9, Invitrogen), h/m IL-5 (TRFK5), mIL-13 (eBio13A, Invitrogen), mIFNg (XMG1.2), and mIL-17A (TC11-18H10.1) in Foxp3/Transcription Factor staining buffer (Invitrogen) or Cytoperm permeabilization buffer (BD Biosciences). The absolute numbers of immune cells were determined by reading the samples with Precision Count Beads (Biolegend, 424902) according to the manufacturer’s instructions. Flow cytometry analysis was performed using BD LSRFortessa X-20 (BD Bioscience), Symphony A5 (BD Bioscience), LSRII (BD Bioscience; Core Facility for Cell to In vivo Imaging of Gachon University) and Cytek Aurora (Cytek Biosciences) instruments. The data were analyzed using FlowJo software v10 (BD Biosciences).

### Histology

Kidney tissues were fixed in 4% paraformaldehyde (Biosesang, P2031) at 4 °C for 24 h. The samples were then embedded in paraffin and prepared for hematoxylin and eosin (H&E) and periodic acid-Schiff (PAS) staining. Sections were scanned using Axio Scan Z1 or Axio Scan 7 (Carl Zeiss, Germany) and analyzed with Zen Blue software (Carl Zeiss) to evaluate kidney histology and quantify the glomerular size. The glomerular size was calculated as the average of 30 glomeruli in each sample.

### Quantitative real-time PCR

Total RNA was extracted from mouse kidney tissues using TRIzol reagent (Invitrogen, 15596018), and cDNA was synthesized using an iScript cDNA Synthesis Kit (Bio-Rad, 1708891). RT‒PCR was performed in duplicate using a CFX96^TM^ real-time PCR detection system (Bio-Rad) and a SensiFAST SYBR Lo-Rox kit (Bioline, BIO-94020). The expression levels of genes of interest were normalized to the expression of *Gapdh* and are presented as 2^−ΔΔCt^ values.

### Anti-dsDNA IgG ELISA

Under deep anesthesia with isoflurane inhalation, blood was collected through the retro-orbital sinus with a heparin-coated glass capillary tube (DWK Life Science, 41B2501). The blood was then centrifuged at 14,000 rpm for 10 min to separate the serum. The serum samples were analyzed for anti-dsDNA IgG levels using commercially available kits (Alpha Diagnostics, 5120). Briefly, 20 μl of serum sample was diluted with 80 μl of working sample diluent, and the diluted sample was further diluted with low-NSB sample diluent (Alpha Diagnostics) at a 1:10 ratio. The diluted samples and standards were incubated in precoated plates. After washing, mouse anti-dsDNA IgG was detected using anti-mouse IgG HRP and TMB substrates. The absorbance at 450 nm was obtained using a microplate reader (Sunrise TW, Tecan).

### Blood urea nitrogen (BUN) and creatinine assays

QuantiChrom Urea and Creatinine Assay Kits (BioAssay Systems, DIUR-100 and DICT-500) were utilized to measure the blood urea nitrogen (BUN) and creatinine (Cr) levels according to the manufacturer’s instructions.

### Intravascular immune cell staining

Mice were anesthetized with isoflurane and intravascularly injected with BV650-labeled anti-mouse CD45 monoclonal antibodies (clone: 30-F11, Biolegend) to distinguish immune cells in the renal vasculature from those in the renal parenchyma. This injection was performed 5 min prior to euthanasia according to a protocol described previously [51].

### FTY720 treatment

A 5 mg/ml solution of FTY720 (Sigma, SML0700) was prepared in PBS and stored at −20 °C. Mice were intraperitoneally injected with 20 μg of FTY720 for three consecutive days and immune cell changes were evaluated after 7 days.

### Human blood ILC and mouse kidney ILC preparation

Human blood was obtained in a heparin-containing tube and then diluted in PBS at a 1:2 ratio. The diluted blood was layered on Ficoll-Paque PLUS solution (GE Healthcare, 17-1440-03) and centrifuged at 2000 rpm for 30 min. Peripheral blood mononuclear cells (PBMCs) were obtained from between the Ficoll layer and the diluted sample layers. For in vitro studies of mouse kidney ILC2s, mice were intraperitoneally injected with 400 ng of recombinant mouse IL-33 (BioLegend, 580508) for three consecutive days. After 7 days, the mice were euthanized, and the tissues were harvested. Cells from mouse tissues were processed using the same method as described above. Human blood or mouse tissue cells were treated with the EasySep Human or Mouse Pan-ILC Enrichment Kit (STEMCELL Technologies, 17975 and 19875), and mouse CD45 Microbeads (Miltenyi Biotec, 130-097-658) were applied to mouse cells to enrich for ILCs. The enriched cells were then stained with fluorochrome-conjugated monoclonal antibodies to identify ILC2s. For the adhesion assay described below, ILC2s were sorted using a FACSAria III sorter (BD Biosciences) and stored in complete RPMI 1640 medium supplemented with 10% FBS until use in subsequent procedures.

### Adhesion assay and live-cell imaging

The ability of the sorted human and mouse ILC2s to adhere to various ligands was examined. The cells were incubated at 37 °C until imaging. The 6-well plates were precoated with 0.1 μM MAdCAM-1 (R&D Systems, 993-MC-050), VCAM-1 (R&D Systems, 643-VM-050), E-cadherin (Biolegend, 780004), or fibronectin (Sigma, F1141) for 1 h at room temperature. After washes with PBS, the wells were blocked with 5% BSA for 1 h. For live-cell imaging, the sorted ILC2s were loaded onto the prewarmed stage, and the images were recorded for 30 to 60 min at 30-s intervals at 60x magnification. The cells were incubated on ligand-coated plates for 1 h at 37 °C and washed three times with PBS to compare the ligand-binding profiles. The adherent cells were fixed with 4% paraformaldehyde (Biosesang), and images of the fixed cells were acquired. Imaging was performed using an Eclipse Ti microscope (Nikon) equipped with a live imaging chamber that maintained the cells under physiological conditions (37 °C, 5% CO_2_). The acquired images were analyzed using acquisition software (Nikon).

### Downregulation of integrin expression using morpholino oligonucleotides (MOs)

Kidney ILC2s were cultured and expanded in complete RPMI 1640 medium supplemented with 10 ng/ml IL-2 (BioLegend, 575404), 10 ng/ml IL-7 (BioLegend, 577804), and 30 ng/ml IL-33 (BioLegend, 580508). Two days prior to incubation with morpholinos, the ILC2s were maintained in medium containing only 10 ng/ml IL-2 and IL-7. For the experiment, 1.0 × 10^4^ ILCs or sorted CD4 T cells were incubated with 2.0 μM or 4.0 μM Vivo morpholino (Gene Tools) specifically targeting *Itga4* or with random control oligos in maintenance medium for 48 h. The oligo sequence used to target *Itga4* was designed by Gene Tools.

### Effect of Toll-like receptor (TLR) agonist treatment on ILC2s

Kidney ILC2s were prepared in expansion media composed of 10 ng/mL IL-2, 10 ng/mL IL-7, and 30 ng/mL IL-33 in RPMI 1640 medium supplemented with 10% FBS. After 48 h of incubation without IL-33, kidney ILC2s were treated with TLR agonists (5 µg/ml poly(I:C), 200 ng/ml LPS, 1 μg/ml ssRNA40, and 4 μM ODN1826) from a mouse TLR1-9 agonist kit (Invitrogen, tlrl-kit1m2) in IL-2- and IL-7-containing complete RPMI 1640 medium for 48 h. The expression of integrins was evaluated using flow cytometry or RT‒PCR.

### Single-cell RNA sequencing

Single cells were isolated from the pooled kidneys of old or young *lpr* mice after leukocyte enrichment via density gradient centrifugation. Single-cell suspensions of kidney tissue were further enriched using a mouse pan-ILC enrichment kit (STEMCELL Technologies). Dead cells were stained and excluded using Zombi-Aqua dye (Biolegend), and the live cells (Zombi-Aqua^neg^ cells) were sorted using a FACSAria III sorter (BD Biosciences). RNA was extracted from 10,000 sorted live cells, and cDNA libraries were prepared using 10x Chromium single-cell 3′ reagent kits v3.1 (10x Genomics, 1000121). The libraries were sequenced using the Illumina NovaSeq 6000 platform with paired-end 100 bp reads, targeting an average of 50,000 read pairs per cell. The Cell Ranger v3 program (10x Genomics) was used for demultiplexing, alignment (the mouse mm10 reference genome), and quantification of the sequenced data to generate barcode matrices. The downstream processing and analysis of the data were performed using the R package Seurat v4.1.0.

The quality control of the processed matrix was performed by filtering out cells with a >20% mitochondrial gene ratio and/or <200 features. The data were log-normalized using the log normalization method with a scale factor of 10000. The highly variable features (nfeatures = 2000) were identified using the FindVariableFeatures() function with the vst method. Each object was integrated with the FindIntegrationAnchors() and IntegrateData() functions at a ratio of 1:20 to study the single-cell data from the control kidneys and lupus nephritis kidneys. The scaled data were further used for dimensionality reduction via the RunPCA() function. Clusters were identified using 10 principal components via the FindNeighbors() and FindClusters() functions and projected on a uniform manifold approximation and projection (UMAP) plot via the RunUMAP() function. Marker gene expression in the clusters was used to annotate the types of the cell clusters. The interacting partners of the integrin-expressing ILC2s were inferred using SingleCellSignalR (v1.4.0). The physiological interaction of kidney ILC2s in the homeostatic kidneys was estimated based on data from young MRL-*lpr* mice. With a threshold LR score of 0.5 and logFC score of 1.5, the interaction was predicted using the paracrine and autocrine options to include both exclusive and partial receptor‒ligand combinations between the different cell types. Single-cell RNA sequencing data were deposited in GEO (accession number GSE234742).

### Statistical analysis

The data are shown as the means ± standard errors of the means. The data were analyzed using the Mann‒Whitney *U* test for two-group comparisons or the Kruskal‒Wallis test followed by Dunn’s post hoc test for multiple group comparisons. The correlation analysis was conducted with Spearman’s correlation test. A *P* value less than 0.05 was considered to indicate statistical significance. All the statistical analyses were performed using GraphPad Prism 10 software (GraphPad).

### Study approval

Ethical approval for all animal experiments was obtained from the Seoul National University Hospital Institutional Animal Care and Use Committee (17-0062 and 20-0036) and Gachon University Lee Gil Ya Cancer and Diabetes Institutional Animal Care and Use Committee (LCDI-2023-0117). All tissues and blood samples from volunteers were collected after obtaining written informed consent from the participants. The study using human samples was approved by the Seoul National University Hospital Institutional Review Board (IRB number 1709-105-887).

### Supplementary information

Supplementary figure revised

Supplementary Movie S1

Supplementary Movie S2

Supplementary Movie S3

Supplementary Movie S4

## Data Availability

Single-cell RNA sequencing data were deposited in GEO (accession number GSE234742) and are publicly available. Any additional information required to reanalyze the data reported in this paper is available from the corresponding author upon request.
