# Supplementary material for: The protective roles of integrin α4β7 and Amphiregulin-expressing innate lymphoid cells in lupus nephritis
Source: Cell Mol Immunol. 2024 May 28;21(7):723–37. doi: 10.1038/s41423-024-01178-2 (PMC11214630; doi:10.1038/s41423-024-01178-2)
Supplement: Supplementary file 1 — Supplementary figure revised [file 41423_2024_1178_MOESM1_ESM.doc]

**Supplementary figure legends**

**
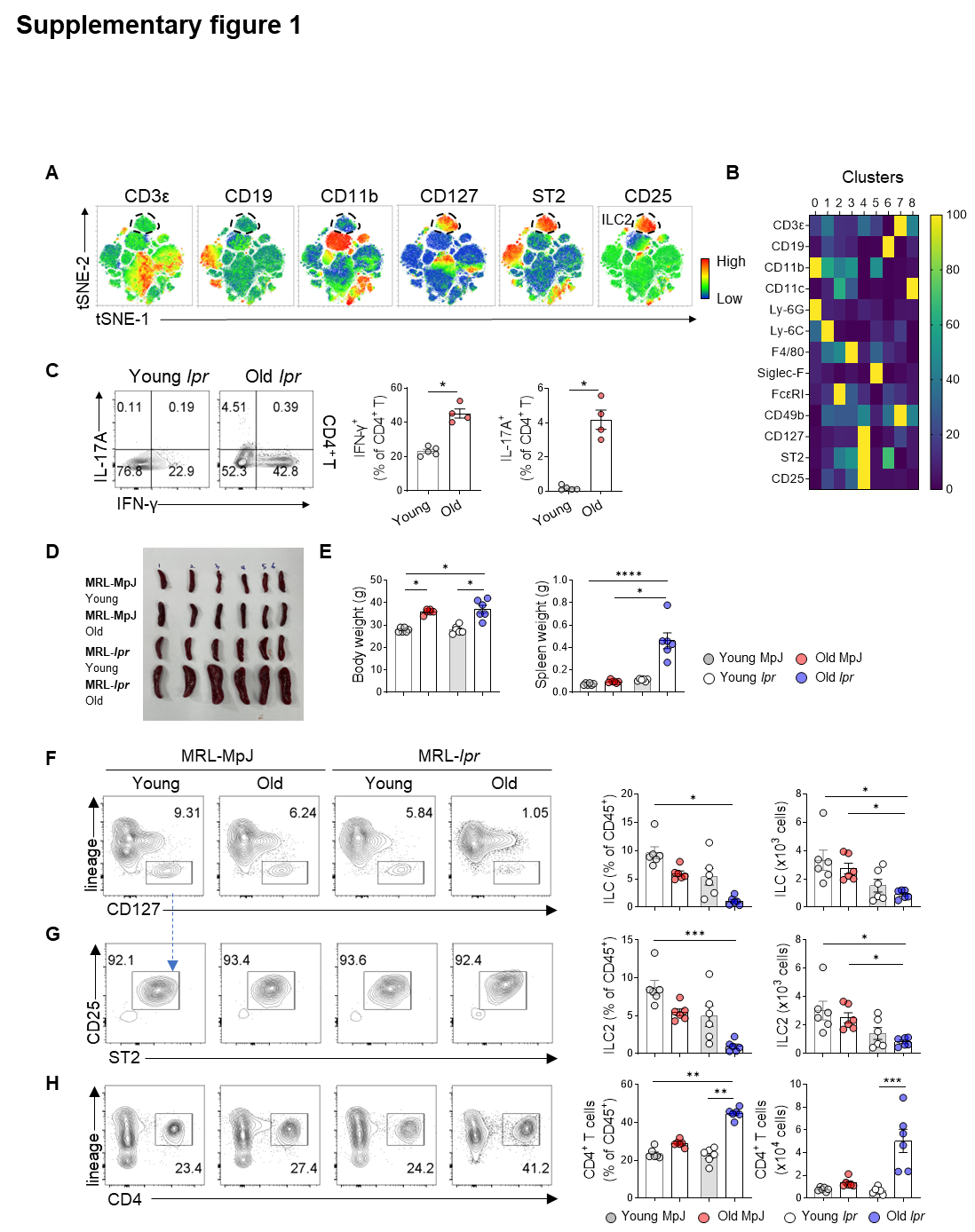
**

**Supplementary figure 1. The spontaneous development of lupus in MRL-*lpr* mice associates with reduced kidney ILC2 numbers, related to Figure 1.**

(A) t-SNE plot of flow cytometry data showing the expression of ILC2-related surface markers generated by ClusterExplorer, a FlowJo plugin. (B) Heatmap of the expression of representative surface markers for immune cell subsets in the kidney with their relative expression in each cluster. (C) Percentage of IFN-g and IL-17A-expressing CD4^+^ T cells in MRL-*lpr* kidney (n= 4-5). (D) Body and spleen weight of young and old MRL-MpJ and MRL-*lpr* mice (n = 6 for each group). (F-H) Frequency and absolute numbers of kidney ILCs (F), ILC2s (G) and CD4^+^ T cells (H). All results are shown as mean ± SEM, and statistical analysis was performed using Mann-Whitney *U* or Kruskal-Wallis test. **P*<0.05; ***P*<0.01; ****P*<0.001.

**
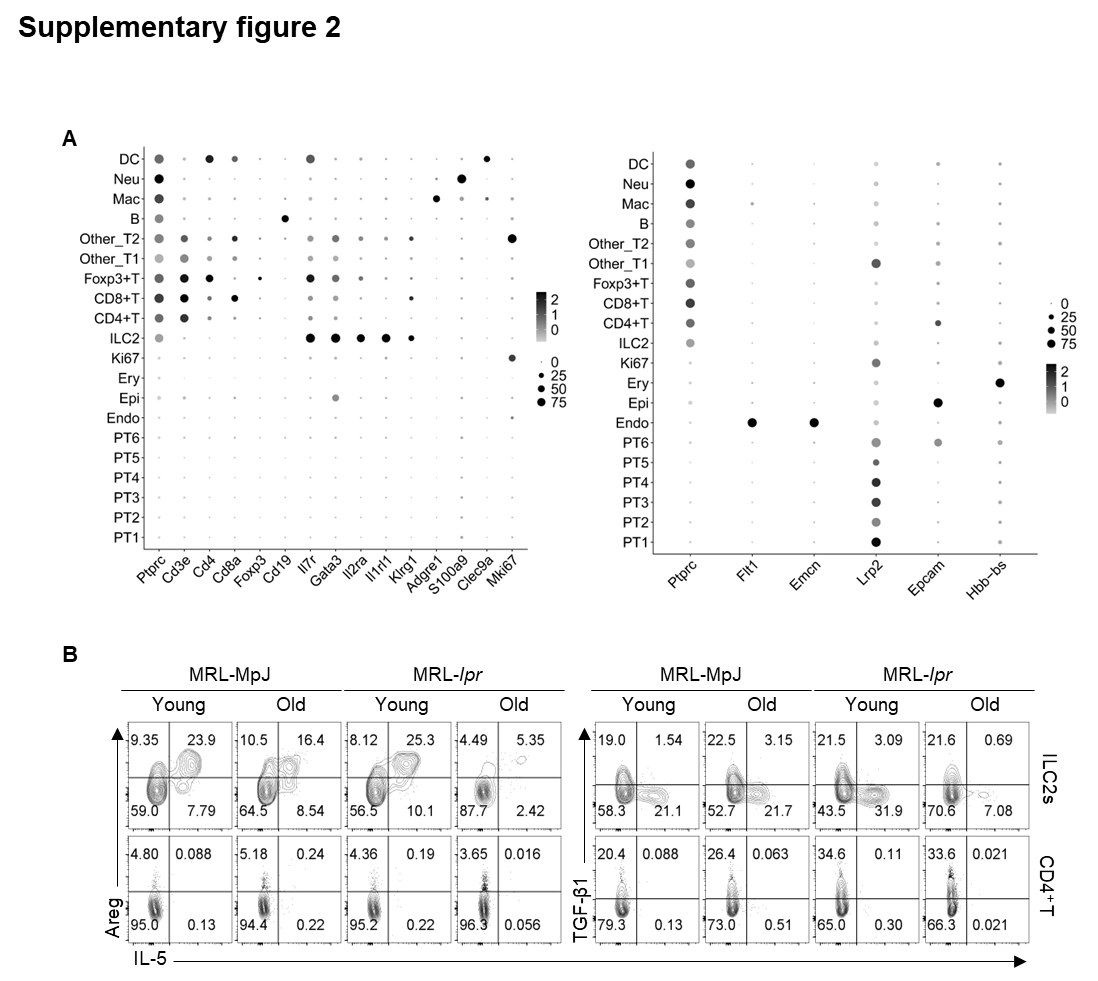
**

## Supplementary figure 2. The spontaneous development of lupus in MRL-*lpr* mice associates with reduced kidney ILC2 numbers, related to Figure 1.

(A) Dot plot showing expression of marker genes for immune (left) and non-immune cells (right) for the clusters of scRNA-seq data. (B) Expression of Areg, TGF-β1, and IL-5 in ILC2s and CD4^+^ T cells from MRL-MpJ and MRL-lpr kidney.


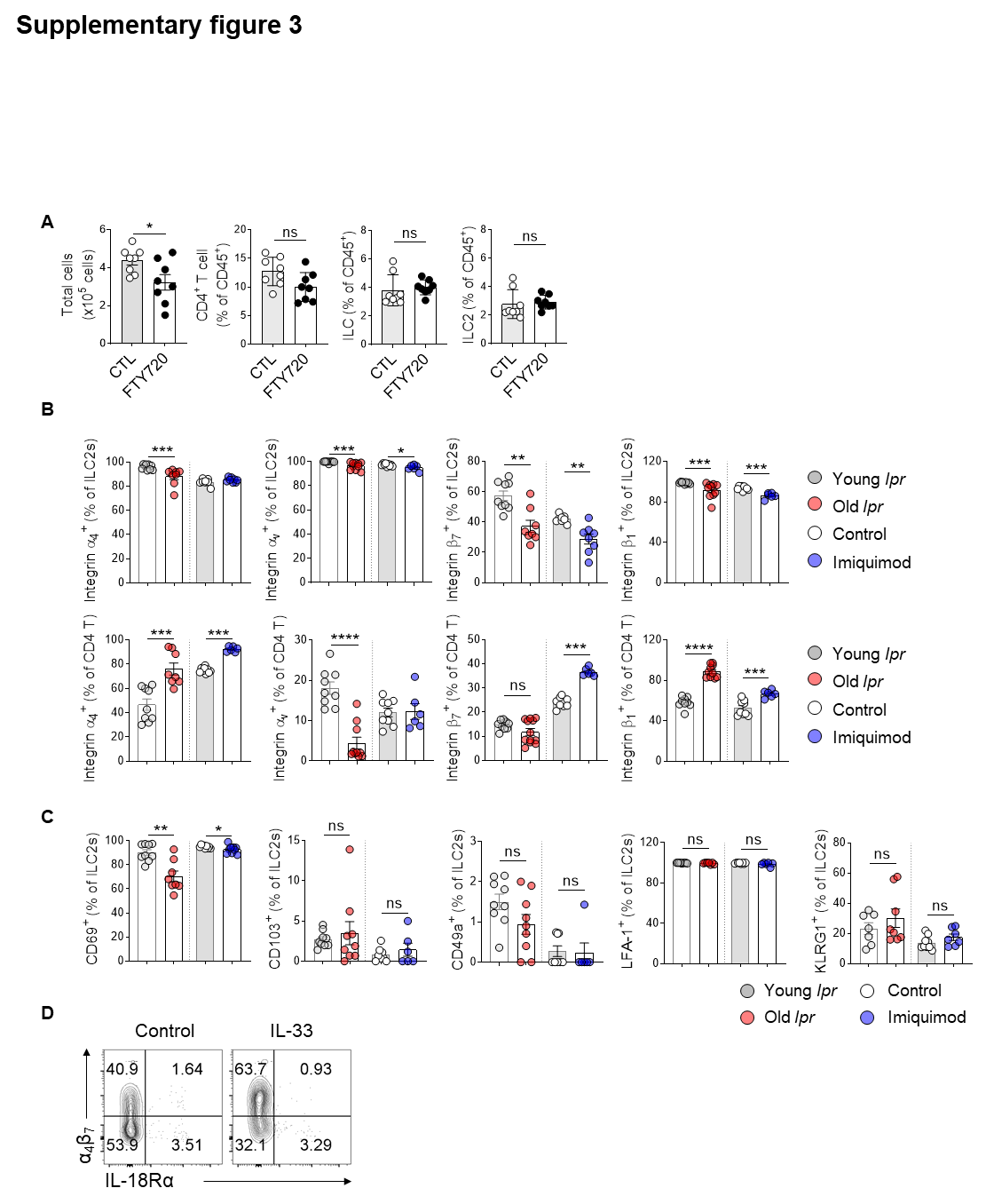


## Supplementary figure 3. Kidney-resident ILC2s express high levels of integrin α4β7 and lupus nephritis associates with the loss of this expression, related to Figure 3.

(A) Total renal cell numbers, and the frequency of CD4^+^ T cells, ILCs, and ILC2s by FTY720 treatment (n = 8). (B) Frequencies of integrin α4, αv, β7, and β1 in kidney ILC2s and CD4^+^ T cells from MRL-lpr and IMQ lupus model (n = 6-12). (C) Frequencies of CD69, CD103, and CD49a in kidney ILC2s from MRL-lpr and IMQ lupus model (n = 6-9). (D) Expression of integrin α4β7 and IL-18Rα in kidney ILC2s by systematic treatment of the recombinant IL-33 protein. All results are shown as mean ± SEM, and statistical analysis was performed using Mann-Whitney U test. ns, no significance; *P<0.05; **P<0.01; ***P<0.001; *****P*<0.0001.

**
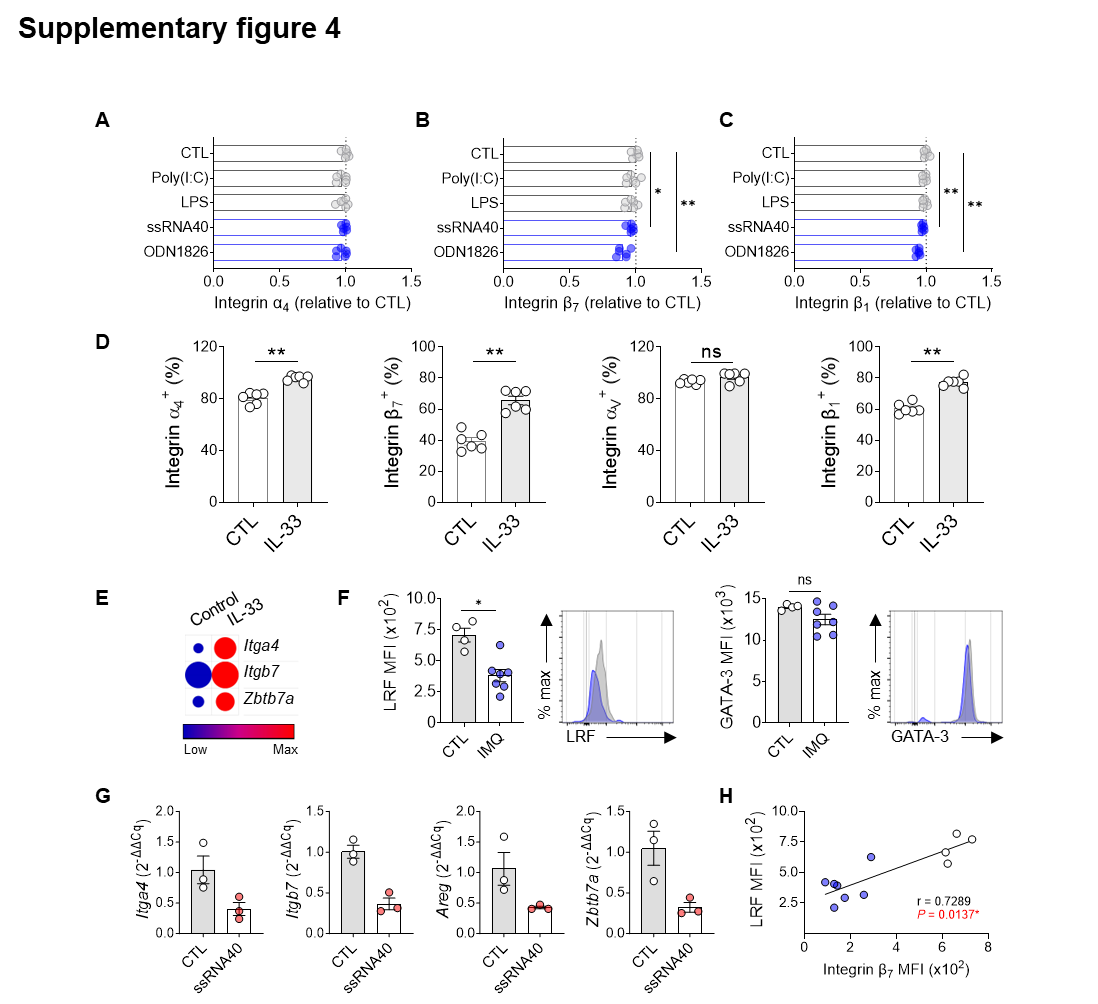
**

**Supplementary figure 4. TLR7 and TLR9 signaling reduces the integrin expression of kidney ILC2s, whereas IL-33 stimulation increases it, related to Figure 6.**

(A-C) Relative expression of integrin α4 (A), β7 (B), and β1 (C) in kidney ILC2s in response to TLR agonists for 48 hours, including Poly(I:C), LPS, ssRNA40, and ODN1826 (n = 6), evaluated by flow cytometry. (D) Expression of integrin α4, β7, αv, β1 was compared in the recombinant IL-33 treated kidney ILC2s (n = 6). (E) Expression of *Itga4*, *Itgb7*, and *Zbtb7a* by IL-33 evaluated by Broad Institute - Single Cell Portal (https://singlecell.broadinstitute.org/single_cell) using data from Wallrapp et al [54]. (F) LRF and GATA-3 expression evaluated by flow cytometry in IMQ-induced model (n = 4-7). (G) Expression of *Itga4*, *Itgb7*, *Areg*, and *Zbtb7a* in lung ILC2s in response to ssRNA40 for 48 hours (n = 3). (H) Correlation between integrin β7 and LRF expression evaluated by flow cytometry in IMQ-induced model (n = 4-7). All results are shown as mean ± SEM, and statistical analysis was performed using Mann-Whitney U test or Spearman’s correlation test. ns, no significance; *P<0.05; **P<0.01.

**
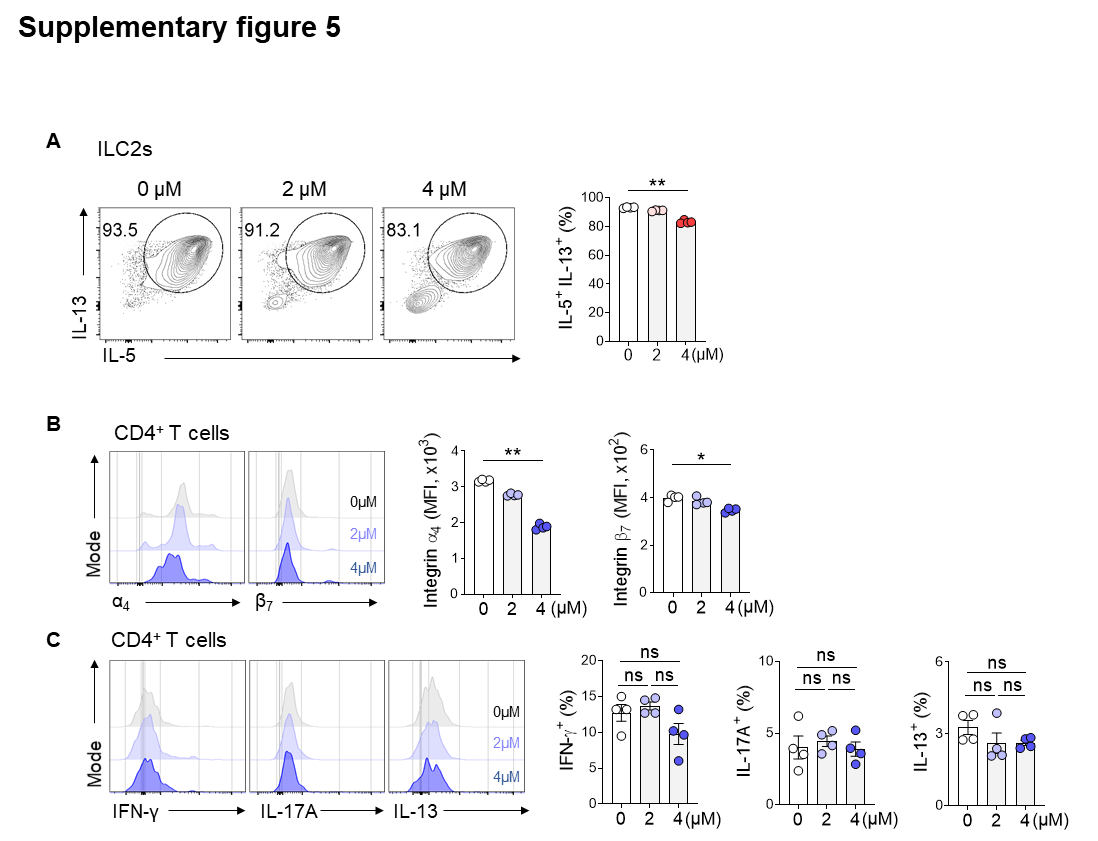
**

## Supplementary figure 5. Integrin-α4β7 knockdown in kidney ILC2s impairs cytokine expression, related to Figure 6.

(A) Expression of IL-5 and IL-13 in the kidney ILC2s by MO Itga4-mediated downregulation (n = 4). (B) Expression (MFI) of integrin α4 and β7 by MO Itga4 in kidney CD4^+^ T cells (n = 4). (C) Frequencies of IFN-γ, IL-17A, and IL-13 by MO Itga4 in kidney CD4^+^ T cells (n = 4) All results are shown as mean ± SEM, and statistical analysis was performed using Kruskal-Wallis test. ns, no significance; *P<0.05; **P<0.01.

**
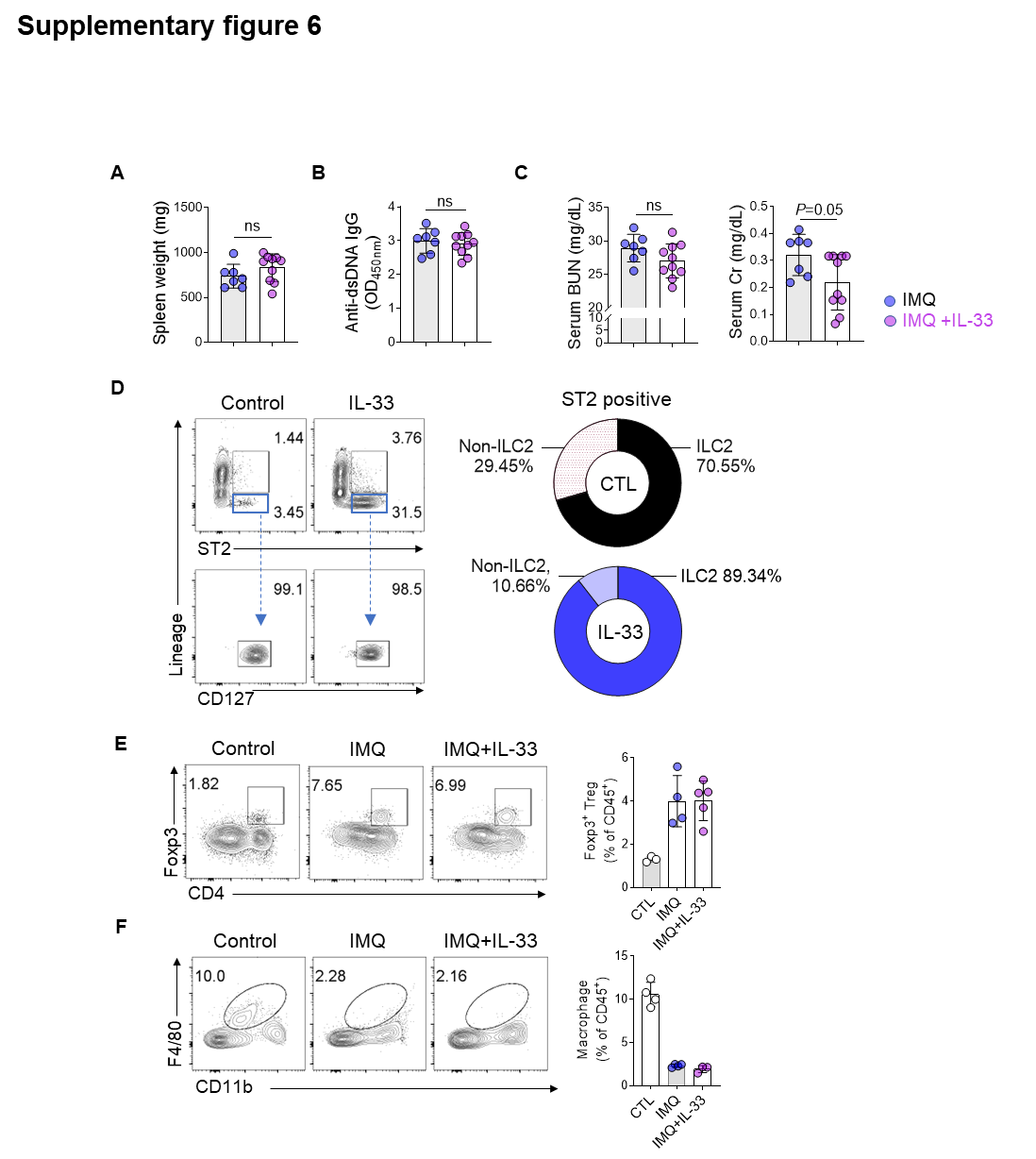
**

**Supplementary figure 6. IL-33 mediated expansion of kidney-resident ILC2s improves survival and renal function in lupus nephritis, related to Figure 7.**

(A-C) Spleen weight (A), serum anti-dsDNA IgG (B), BUN, and Cr levels (C) of IL-33 treated IMQ-model (n = 7-10). (D) Comparison of ST2-expressing immune cells in control and IL-33-treated kidneys (n = 3-5) (E-F) Frequencies of Foxp3^+^ regulatory T cells (Treg) (E) and macrophages (F) in the control and IMQ lupus mice by the recombinant IL-33 treatment (n = 3-5). All results are shown as mean ± SEM, and statistical analysis was performed using Mann-Whitney U test. ns, no significance.


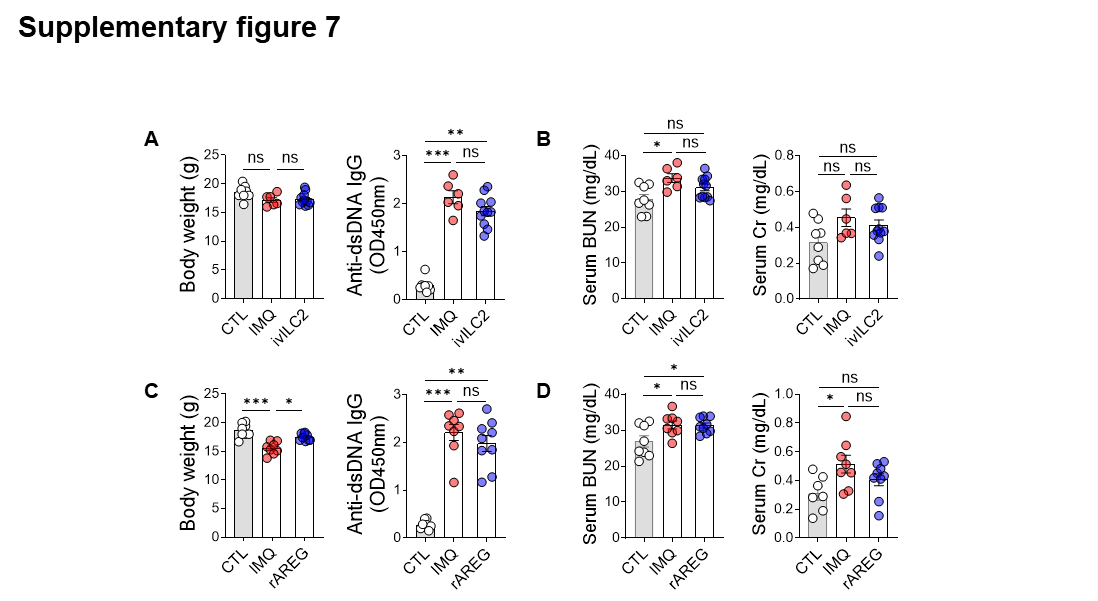


**Supplementary figure 7. IL-33 mediated expansion of kidney-resident ILC2s improves survival and renal function in lupus nephritis, related to Figure 7.**

(A, C) Body weight and serum anti-dsDNA IgG levels by (A) ILC2 adoptive transfer and (C) rAREG administration in the IMQ-induced model (n = 6-11). (B, D) Serum BUN and Cr levels by (B) ILC2 adoptive transfer and (D) rAREG administration. All results are shown as mean ± SEM, and statistical analysis was performed using Kruskal-Wallis test. ns, no significance; **P*<0.05; ***P*<0.01; ****P*<0.001.

**Supplementary movie file legends**

Supplementary Movie 1 (mp4 format). Live-cell imaging of kidney ILC2s on the MAdCAM-1 coated plate.

Supplementary Movie 2 (mp4 format). Live-cell imaging of kidney ILC2s on the VCAM-1 coated plate.

Supplementary Movie 3 (mp4 format). Live-cell imaging of kidney ILC2s on the E-cadherin coated plate.

Supplementary Movie 4 (mp4 format). Live-cell imaging of kidney ILC2s on the fibronectin coated plate.
